# Finite element modeling of clavicle fracture fixations: a systematic scoping review

**DOI:** 10.1007/s11517-025-03294-1

**Published:** 2025-01-28

**Authors:** Yi Zheng, Jing Li, Andy Yiu-Chau Tam, Timothy Tin-Yan Lee, Yinghu Peng, James Chung-Wai Cheung, Duo Wai-Chi Wong, Ming Ni

**Affiliations:** 1 Department of Biomedical Engineering, Faculty of Engineering, The Hong Kong Polytechnic University, GH140, 1/F, GH Wing, 11 Yuk Choi Road, Hung Hom, Hong Kong, China; 2 School of Electrical and Electronic Engineering, Nanyang Technological University, Singapore, Singapore; 3 Laboratory of Human-Machine Intelligence – Synergy Systems, Shenzhen Institutes of Advanced Technology, Chinese Academy of Sciences, Shenzhen, China; 4 Research Institute for Sports Science and Technology, The Hong Kong Polytechnic University, Hong Kong, China; 5 Department of Orthopaedics, School of Medicine, Ruijin Hospital, Shanghai Jiao Tong University, Shanghai, China; 6 Laboratory of Prevention and Treatment of Bone and Joint Diseases, Shanghai Institute of Traumatology and Orthopaedics, Ruijin Hospital, School of Medicine, Shanghai Jiao Tong University, Shanghai, China

**Keywords:** Computational model, In silico, Shoulder, Clavicle fracture, Biomechanics, Surgery

## Abstract

Finite element analysis has become indispensable for biomechanical research on clavicle fractures. This review summarized evidence regarding configurations and applications of finite element analysis in clavicle fracture fixation. Seventeen articles involving 22 clavicles were synthesized from CINAHL, Embase, IEEE Xplore, PubMed, Scopus, and Web of Science databases. Most studies investigated midshaft transverse closed fractures by reconstructing intact models from CT scans and simulating fractures through gap creation. Common loading schemes included axial compression, distal torsion, and inferior bending. The primary objective was comparing different implant designs/placements on construct stiffness, von Mises stress, and fracture site micro-motion. Our review suggested a preference for plate fixation, particularly with anterior placement, for midshaft transverse fractures. However, limited fracture types studied constrain comprehensive recommendations. Additionally, the review highlighted discrepancies between finite element and clinical studies, emphasizing the need for improved modeling of physiological conditions. Future research should focus on developing a comprehensive database of finite element models to test various implant options and placements under common loading schemes, bridging the gap between biomechanical simulations and clinical outcomes.

**Graphical Abstract:**

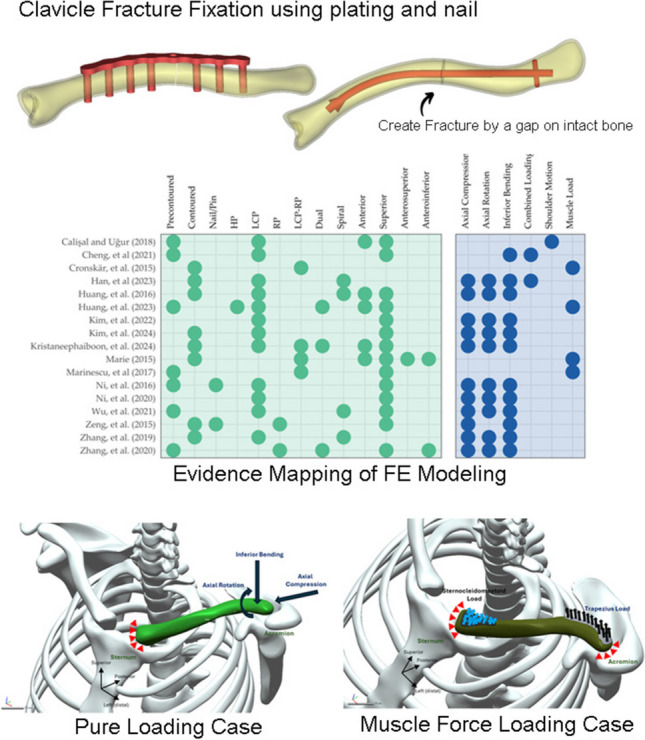

**Supplementary Information:**

The online version contains supplementary material available at 10.1007/s11517-025-03294-1.

## Introduction

Clavicle fractures are common injuries, predominantly caused by high-energy trauma, such as falls, collision sports, and motor accidents [1]. The incidence rate was 22.4 per 100,000 person-years, with athletic activities contributing to half of these cases [2]. Another study reported that athletes had an annual clavicle fracture rate of 18.72 per 10,000 individuals at risk [3]. An analysis of a healthcare claim database revealed that patients who underwent surgery incurred a cost of US$27,635 over a 2-year follow-up period [4]. While a clavicle fracture can cause pain and limited shoulder mobility and significantly impact daily living [5], it often occurs alongside more severe injuries such as hemopneumothorax, diaphragm injury, and fractures of the humerus and ribs [6].

Conservative treatments, such as shoulder-arm sling and figure-of-eight bandage, are often recommended, particularly for pediatric patients [7, 8]. However, in cases of serious injury, characterized by displacement, comminution, or associated neurovascular issues, surgical interventions are necessary [9, 10]. Surgical options include plate fixation, intramedullary nailing, external fixation, and open reduction and internal fixation (ORIF). Surgical interventions might involve plate fixation, intramedullary nailing, and external fixation [9, 11]. Despite this, the complication rates for surgeries remain high, with infection, non-union, and malunion rates reported at 1.0%, 4.2%, and 0.9%, respectively [12]. Other studies indicated that the non-union rate of ORIF was 2.6%, but this rate can increase to as much as 5.9% with conservative treatments and exceeds 15% for conservative treatments on displaced fractures [13, 14]. Charles et al. [15] reported an overall complication rate of 27.3% with 9.1% requiring a re-surgery. Complications after clavicle fracture fixation are undeniable.

While age, bone quality, and lifestyle are associated with the risk of complications [1, 6], surgical techniques and implant-specific factors play a significant role [16]. A meta-analysis estimated that intramedullary fixation, particularly using titanium elastic nail, had a pooled incidence of 20% for hardware irritation, and 12% for either protrusion, telescoping, or migration [16]. Besides, another study reported an implant failure rate of 4% [17]. More than one-tenth of patients required hardware removal and 1% needed reoperation [18]. The choice and positioning of implants could impact complication and reoperation rates [15, 19, 20]. Superior plating has been associated with increased irritability, while some studies have indicated that anteroinferior plating leads to fewer postoperative symptoms [14, 21]. Moreover, more screws and openings on the locking plates could substantially reduce the strength of the implant [22]. Improved implant designs, such as pre-contoured plates, could reduce non-union rates [18]. While it is challenging to conduct rigorous clinical studies for clavicle fracture fixation [23], biomechanical studies can provide information on implant and bone stress, thereby uncovering the mechanisms of complications and offering evidence to guide implant design and surgical decisions.

Mechanical testing using cadaveric experiment is a widely employed methodology in biomechanical research [24]. Kitzen et al. [25] investigated the axial, torsional, and bending stiffness, as well as load-to-failure and 3-point superior surface bending strength, comparing pre-contoured superior and anterior fixation methods for inferior butterfly clavicle fractures. Rieser et al. [26] assessed the construct stiffness and stability by comparing locking plates, acromioclavicular tightropes, and their combination for distal clavicular fractures. To address the scarcity and ethical concerns related to cadaveric experiments, synthetic bones (commonly referred to as sawbones) were also utilized. Partal et al. [27] conducted a similar study to Kitzen et al. [25] but employed sawbones to discern differences in axial and torsional stiffness between plates positioned superiorly and anteroinferiorly. Moreover, Worhacz et al. [28] loaded sawbones simulating a 45° arm abduction with 2 kg weight, comparing straight and pre-contoured plates with locking or non-locking screws. These cadaveric and sawbone studies yielded mixed results, possibly due to variations in test settings and specimens. Furthermore, computational (in silico) methods, particularly finite element method, have emerged, offering a cost-effective approach to study complex loading conditions.

The finite element method is a numerical technique used to solve complex engineering problems and approximate solutions [29]. It is particularly useful in biomechanical investigations where the geometry, loading, and material properties are too complex for an analytical solution. The fundamental principle involves dividing the complex model into smaller units, known as finite elements, which are interconnected by nodes or controlled by boundaries [30]. These elements are reassembled into a global stiffness matrix for the entire model. Each element contains its own stiffness matrix, derived from the material properties, geometry, and boundary conditions. The matrix represents the relationship between the applied load and the deformation. When a load is applied or transferred through other elements, the mathematical equation of the stiffness matrix is solved to determine the nodal displacements of the element. Subsequently, the stress within the element can be calculated using the nodal displacement and the material’s constitutive equation, typically represented by Hooke’s Law. In the context of static simulation using an implicit solver, an iterative approach is implemented to update the nodal displacement until equilibrium is reached between the internal force and the applied (external) load. The process continues until the solution converges to a specified tolerance [31].

Finite element methods have been widely employed to investigate the biomechanics of the musculoskeletal system, with a particular focus on the spine [32, 33], hip [34], knee [35], and foot and ankle [36, 37]. Applications include surgical and implant design [38, 39], footwear, brace, protective equipment, and interfacial design [40–43]. Nevertheless, in addition to single-subject and patient-specific, the study design of finite element methods often requires a set of pre-set model configuration and a specific simulated scenario [44]. Therefore, it is desirable to summarize existing research to make robust and comprehensive translation to clinical impact. To date, we could not find a relevant review on this topic. The overall objective of this systematic review is to synthesize current research on finite element analysis of clavicle fracture fixation to address the following contextual and conceptual review questions.

Contextual review questions (based on the modeling and technical scope):

- What are the common numerical modeling techniques used in finite element analysis of clavicle fracture fixation?
- Which materials and their properties are most frequently modeled for the bones, soft tissues, and implants in the numerical simulation?
- What loading schemes are applied? Are they related to physiological conditions and real-world scenarios?
- How is model validation and verification typically conducted to ensure the accuracy and reliability of the simulation?

Conceptual review questions (based on the clinical implication and study design scope):

- What are the primary research interests (independent factors) outlined in the numerical analysis of clavicle fracture fixation?
- What outcome measures (dependent factors) are commonly used to evaluate the biomechanics of clavicle fracture fixation?
- What are the key findings and clinical/design implications?

## Search methods

### Search strategy

The protocol of the literature search followed the Joanna Briggs Institute (JBI) scoping review methodology [45, 46] and Preferred Reporting Items for Systematic Review and Meta-Analysis Protocols Extension for Scoping Reviews (PRISMA-ScR) guidelines [47]. The search was conducted on 29 Mar 2024 without any limitation on the year of publication. The first (Z.Y.) and second author (J.L.) independently conducted the literature search and screen on online databases, including CINAHL via EBSCOhost (field: default; filter: English), Embase via OVID (field: title/abstract/keywords; filter: English), IEEE Xplore Digital Library (field: all metadata), PubMed (field: title/abstract; filter: English), Scopus (field: title/abstract/keywords; filter: English), and Web of Science (field: topic; filter: English). Any disagreement was resolved by seeking consensus with the authors.

The keyword, “finite element,” was searched together with the context of clavicle and fixation domains. In the clavicle domain, keywords included “clavicle,” “clavicular,” “collarbone,” “collar-bone,” “acromioclavicular,” and “sternoclavicular.” Keywords of the fixation domain included “surger*,” “fixation*,” “plate*,” “intramedullary,” “nail*,” “implant*,” and “reduction.”

### Screening and selection process

The included paper shall be eligible only if it is an original research article published in English as journal articles (including preprints and in-press papers) or conference full papers. The research should focus on clavicle fracture fixation using 3D (three-dimensional) numerical models and finite element analysis. Other inclusion criteria included: (1) should involve 3D modeling and 3D finite element simulation; (2) should address the clavicle fracture fixation (i.e., the assembly of clavicle and implant models).

Studies were excluded if they (1) were not of the eligible article type, which might be review papers, conference abstracts, book chapters, commentaries, etc.; (2) reconstructed clavicle models without using the medical images of humans, (e.g., sawbones, cadavers, simulated data, and 3D anatomy atlas), but not limited to those referring to existing models reconstructed from medical images of humans; (3) data source of the clavicle model was unknown; (4) sole simulation of either clavicle or implant; and (4) non-solid structural simulations (e.g., fluid simulation and multiphysics simulation).

### Data extraction and analysis framework

The current review framework was informed by the participant-context-concept (PCC) elements and essential items outlined in standard guidelines [45–47]. The subsequent chapter, Results, summarized the study selection process and the sources of eligible articles, followed by the study quality assessment.

Study quality assessment is an essential component of reviews, serving to evaluate the reliability and validity of included studies. For this review, we employed the Methodological Quality Assessment of Single-Subject Finite Element Analysis Used in Computational Orthopaedics (MQSSFE) [44], which was specifically designed for assessing finite element studies in computational orthopedics. This tool is particularly relevant given the unique challenges and requirements of finite element analysis in biomechanical research. The MQSSFE assessment provides valuable insights into the strengths and limitations of the included studies, informing both the interpretation of results and the identification of areas for improvement in future research, which would be integrated into our discussion to address strengths and potential limitations of the reviewed studies.

MQSSFE contains 37 Yes/No questions across six domains, including study design and presentation (items 1 to 8), subject recruitment (items 9 to 12), model reconstruction and configuration (items 13 to 20), boundary and loading conditions, simulation (items 21 to 26), model verification and validation (items 27 to 31), and model assumption and validity (items 32 to 37). It has previously been shown to have sufficient reliability and validity [44]. The assessment was independently conducted by the third and fourth authors, who then consulted with the other authors to achieve consensus in case of any disagreements. The results were expressed as a percentage by the ratio of the “Yes” (or scored) to the total number of question items. They were visualized using a dot plot with pie charts for individual studies, and radar charts combined with pie charts for the aggregated summary.

A summary on the demographics of participants and details of the simulated fractures and implants are provided in Chapter 4. In Chapter 5, in terms of contextual thematic analysis from a modeling and technical perspective [45, 46], we examine the methodologies of modeling, selection of materials, assignment of loading cases and conditions, and the processes of validation and verification. The narrative is illustrated through evidence maps of modeling [48]. From a clinical and study design perspective, conceptual thematic analysis, in Chapter 6, dissects the primary objectives of the articles reviewed, scrutinizes the variants-of-interest, outcomes-of-interest, and their clinical implications, offering a critical analysis. Data visualizations were enabled by the R Studio and Software (R Foundation for Statistical Computing, Vienna, Austria) and an online platform, RAWGraphs [49].

## Search results

### Study selection

**Fig. 1:**
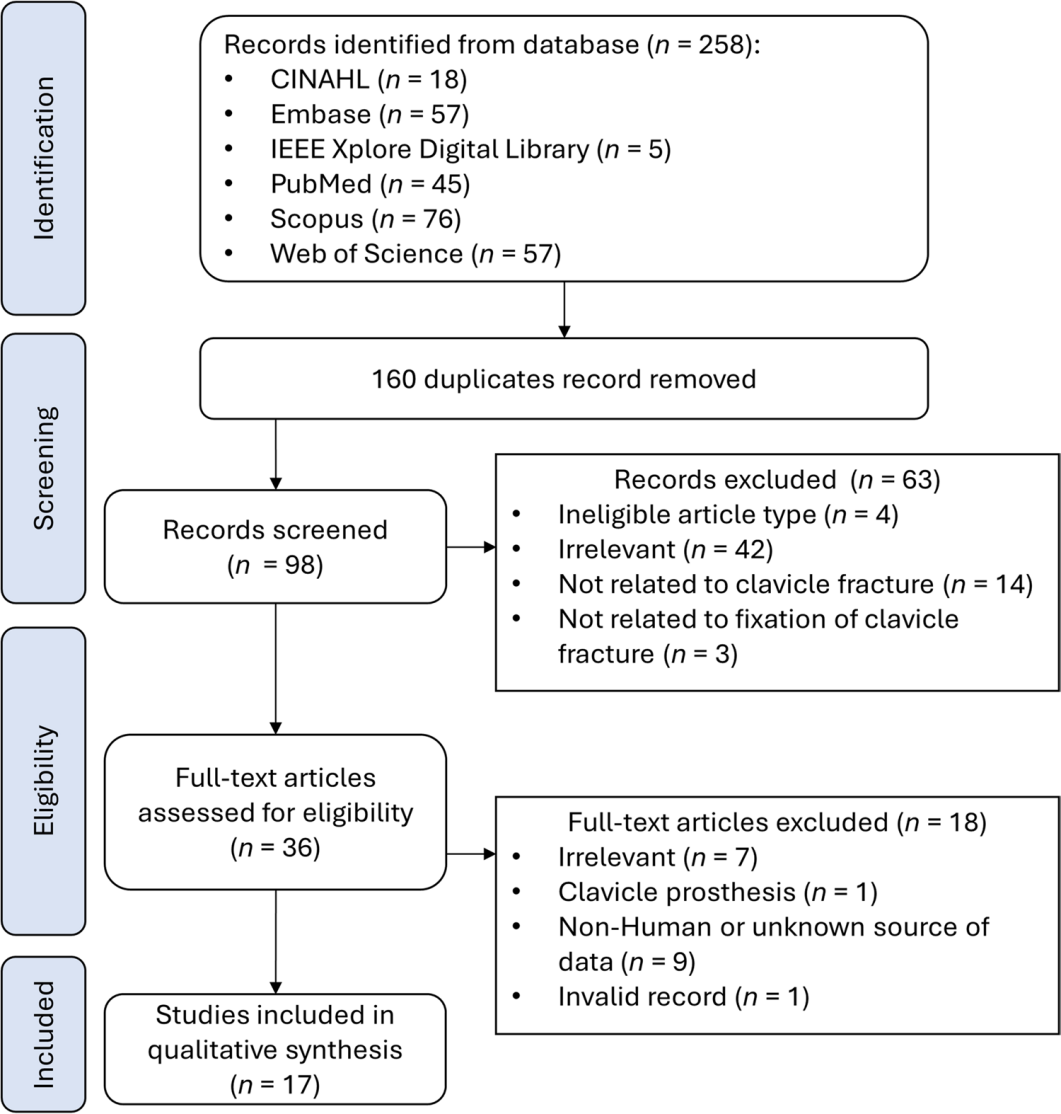
PRISMA flowchart of systematic search and screening

As shown in Fig. 1, we initially identified 258 hits during our search. After removing duplicates, this number reduced to 160 articles. The remaining 98 records underwent preliminary screening based on article type, titles, abstracts, and keywords. We excluded articles for reasons, including ineligibility due to article type (*n* = 4), irrelevance to our main scope, specifically, clavicle and finite element topics (*n* = 42), lack of relevance to clavicle fractures (*n* = 14), and clavicle fracture fixation (*n* = 3). Following this preliminary screening, 35 records remained. Subsequently, we conducted an examination by retrieving the full-text versions. We excluded 18 articles that were irrelevant to our scope (*n* = 7), involved non-human data, or had an unknown data source (*n* = 9). Additionally, one record was deemed invalid (*n* = 1) because it could not be found in the original journal. Lastly, we decided to exclude a paper (*n* = 1) discussing total clavicle replacement with a prosthesis after a discussion. In the end, our review included 17 eligible articles [50–66].

### Evidence source

The papers covered the publication period from 2013 to 2024, and all are journal articles. Six (*n* = 6) originated from the fields of orthopedics and surgery, four (*n* = 4) from biomedical engineering, three (*n* = 3) from interdisciplinary journals, and four (*n* = 4) from other domains. Regarding the country or region of the corresponding authors, Asia contributed the majority of studies, with eight from Mainland China, two from Taiwan, two from the Republic of Korea, and one from Thailand. Europe was represented by four countries: Sweden (*n* = 2), Romania (*n* = 1), and Turkey (*n* = 1).

### Methodological quality assessment

**Fig. 2:**
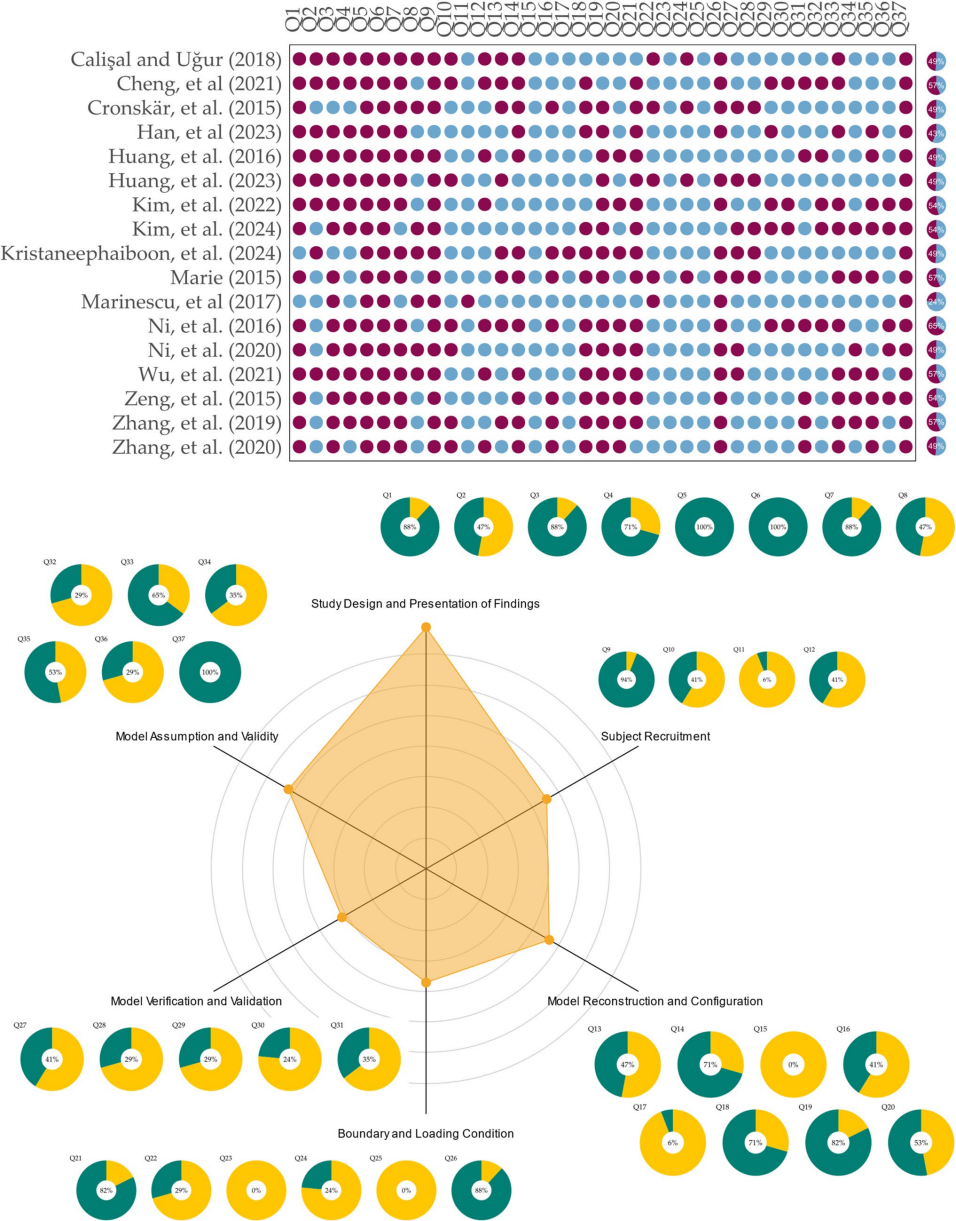
Methodological quality assessment using MQFESS on the reviewed articles. Deep purple color indicates “Yes” (scored) and sky-blue color indicates “No” (not scored) in the dot plot. Data are available in Table S1

The results of the methodological quality assessment conducted using MQFESS for individual studies and their summary are presented in Fig. 2. The average MQFESS score of the reviewed studies was 50.7% (standard deviation, 9%; range, 24.3 to 64.9%). The papers generally performed well in the Study Design and Presentation of Findings, with an average score of 79%. Notably, all studies scored for describing main outcomes and the targeted conditions. Besides, some presentation issues arose, such as unplanned analyses and comparative plots not being presented in the same color scale.

The domain of Model Assumption and Validity received a score of 52%. All studies addressed the implications of their studies and over half discussed the limitations related to the loading scheme and external validity concerns regarding single-subject study. More effort shall be devoted to addressing the limitations associated with modeling and materials, as well as the internal validity and uncertainty inherent in the modeling process. The domains of Subject Recruitment, and Model Reconstruction and Configuration both received a score of 46%. The primary concern for the former was that fractures were simulated rather than derived from actual fractured or postoperative patients, which is challenging given the practical difficulties involved. Noteworthy points included the need for clear details on the reconstruction of the implants, the orientation/posture during image acquisition, and more precise descriptions on the assembly of bones and implants.

Both the Model Verification/Validation and Boundary/Loading Conditions domains performed relatively poor. The former received a score of 32%. Many studies either did not undertake the model verification and validation processes or only completed one of the two. Some studies merely stated that they had completed the process without presenting any results. The latter received a score of 37%. The main issue was that the loading schemes do not physiologically replicate or mimic a daily activity and are arbitrary. Although some studies attempted to simulate the maneuver of drinking coffee, they lack sufficient information to confirm these conditions were driven by the model subject.

## Study information

### Participants

There were 22 participants involved in this review. Among the 17 studies, 16 (*n* = 16) of them adopted a single-subject design, including a total of 8 females and 8 males. The average age in these 16 studies was 41.6 years (standard deviation, 14.8 years; range, 22 to 69). In contrast, Han et al. [53] reconstructed 3D numerical models from 6 healthy participants, comprising 3 males and 3 females, with an age range of 20 to 40 years. Regarding the participant recruitment, 11 studies (*n* = 11) considered healthy participants. Six studies (*n* = 6) recruited patients with clavicle fracture but all of them collected data from the intact side, except for one study that reconstructed a model of postoperative fracture clavicle [60].

### Simulated clavicle fracture

**Fig. 3:**
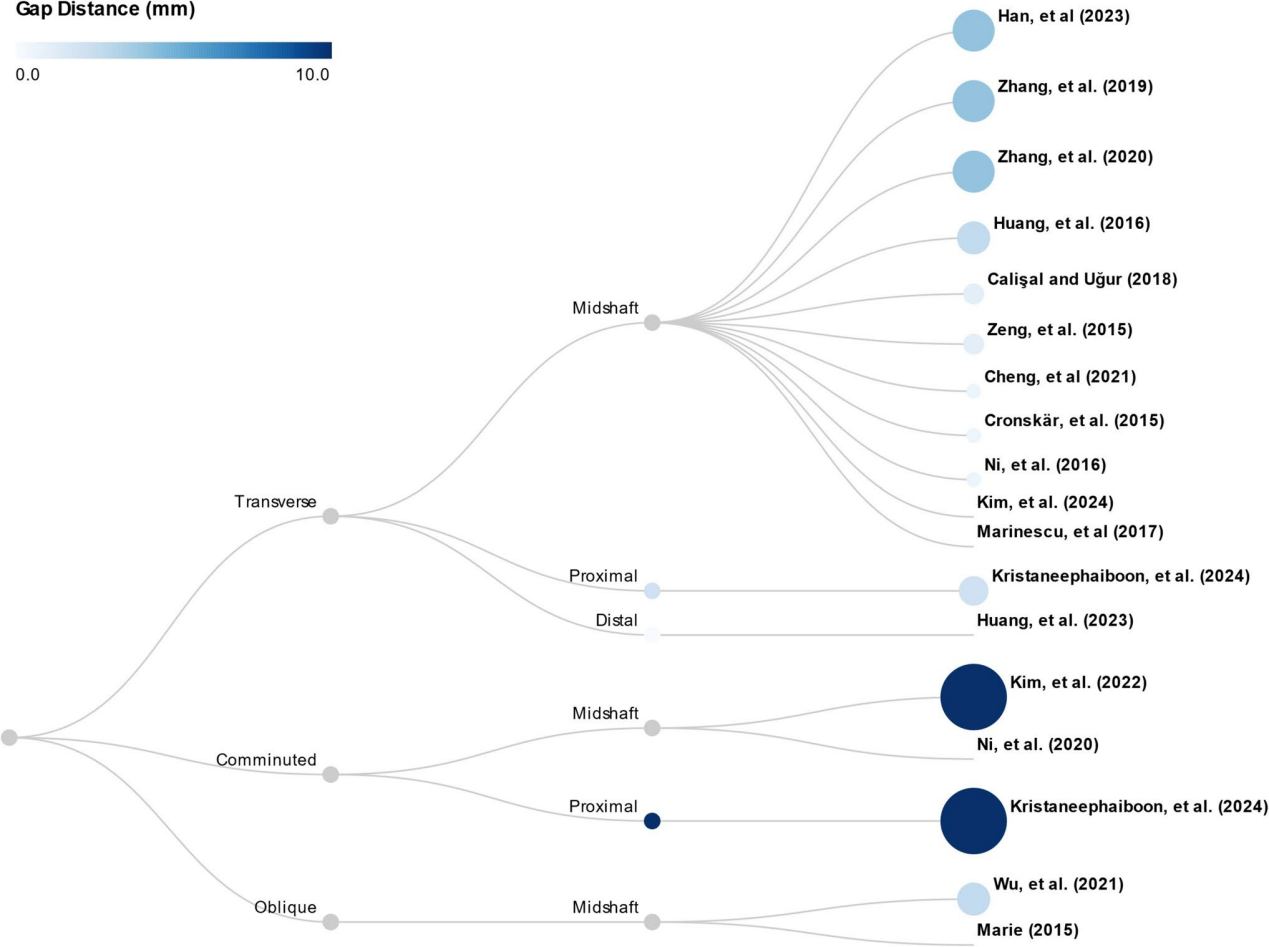
Dendrogram illustrating the clustering of fracture types and locations, in addition to the fracture gap distances (Table S1)

It was reported that 82% of fractures occur in the midshaft and 43% of all clavicle fractures are displaced midshaft fractures [67]. In our review, there were eleven models on midshaft transverse (*n* = 11) [50–53, 55, 57, 60, 61, 64–66], two on midshaft oblique (*n* = 2) [59, 63], one on distal transverse (*n* = 1) [54], and one on proximal transverse fractures (*n* = 1) [58], as shown in Fig. 3 and Table S2. It should be noted that nearly all models reconstructed the clavicle fracture model by reconstructing an intact model and creating a gap. The fracture gap distances for the closed fractures varied between 0.5 and 4.0 mm (Fig. 3 and Table S1). The creation of these fractures from intact models through the introduction of a gap suggested that the distance might be a crucial parameter in characterizing fracture conditions. However, the current approach to determining fracture gap distance appeared arbitrary, with no studies attempting to provide a rationale for their chosen values. Notably, some investigations failed to report these crucial dimensions, despite clear visual evidence of fracture gaps in their accompanying figures.

Three studies/models (*n* = 3) focused on comminuted fractures. Both Kim et al. [56] and Kritsaneephaiboon et al. [58] simulated a large fracture gap of 10 mm, which they assert mimics a comminuted fracture. Conversely, Ni et al. [62] developed a Robinson type 2B1 clavicle fracture model that included the distal and proximal clavicle, with an interposed butterfly fragment.

### Implant designs, configurations, and placements

**Fig. 4:**
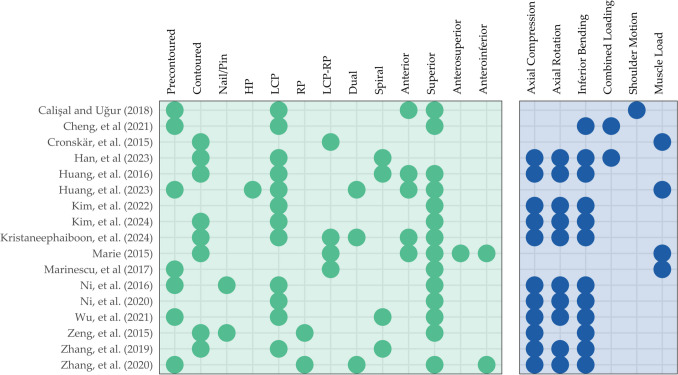
Evidence mapping of modelling on the implant types, configurations and loading cases. HP, hook plate; LCP, locking plate; RP, reconstruction plate

**Fig. 5:**
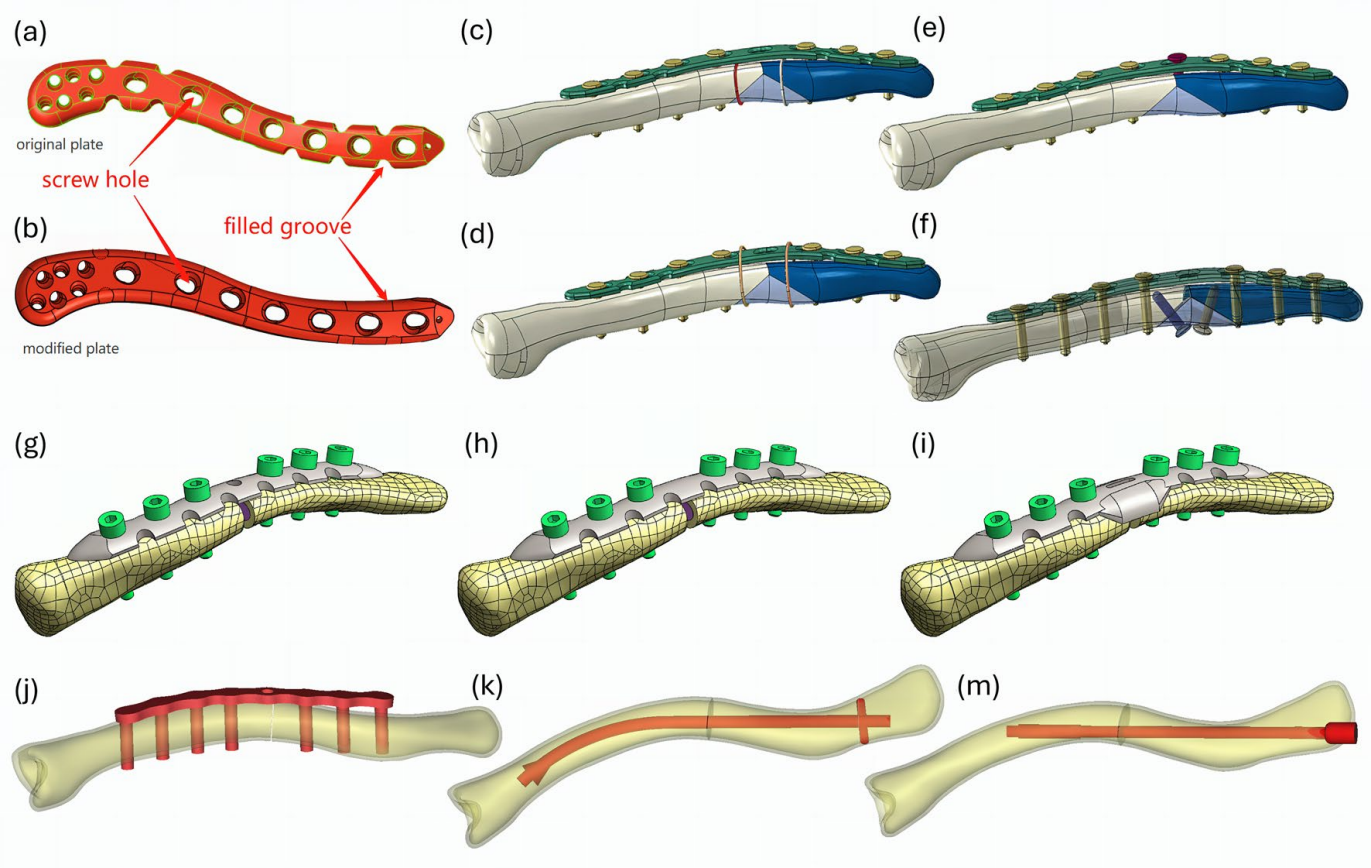
Illustration of models of clavicle fracture fixation in the reviewed articles: **a** modified spiral locking plate [53]; **b** modified spiral locking plate with filled groove and berried central hole [53]; **c** locking plate with double inner cerclage wirings [62]; **d** locking plate with double outer cerclage wirings [62]; **e** locking plate with single interfragmentary screw [62]; **f** locking plate with double interfragmentary screw [62]; **g** customized locking plate [57]; **h** customized locking plate with berried central hole [57]; **i** customized locking plate with double-curved wing [57]; **j** locking plate with locking screws [61]; **k** Sonoma intramedullary nail [61]; **m** Rockwood clavicle pin [61]. (Source: [53, 57, 62] under Creative Commons Attribution License; [61] reprinted with permission from Elsevier Ltd.)

The reviewed articles primarily focused on the design and configuration of implants, as well as their placement, with intramedullary nailing and plate fixation being the two predominant techniques for clavicle fracture fixation (Fig. 4). A meta-analysis showed that the two techniques did not demonstrate a significant difference in long-term functions in fixing displaced fractures [68]. Plate fixation can provide strong and stable fixation for complex fracture, whereas intramedullary fixation, while less invasive, inherits the risk of hardware irritation and protrusion. In our review, two studies (*n* = 2) considered the investigation of intramedullary fixation [61, 64]. Ni et al. [61] compared the performance between the Sonoma intramedullary nail (Sonoma Orthopaedic Products Inc., Santa Rosa, USA) and Rockwood clavicle pin (DePuy Synthes – Johnson & Johnson Medical Devices, West Chester, USA), as shown in Fig. 5. The Sonoma intramedullary nail was anatomically shaped while the Rockwood clavicle pin was straight. Besides, Zeng et al. [64] compared the performance of intramedullary titanium elastic nail (Synthes GmbH, Oberdorf, Switzerland) to the reconstruction plate and intact condition, while Zhang et al. [66] utilized the Herbert fixation.

In the investigation of plate fixations, there were several aspects of interest, including various types of plates, modifications to the plates, and their placements. Occasionally, the terms or categories used to describe them were ambiguous and used interchangeably. Locking plates were frequently investigated, appearing in 12 studies [50, 51, 53–58, 61–63, 66]. They feature screws that secure the plate to the bone, offering better stability. On the other hand, reconstruction plates are straight and offer more flexibility as they can be shaped to match the contour of the bone, albeit with less stability (*n* = 2) [64, 65]. Some studies explored the locking reconstruction plates (i.e., a combination of both types) (*n* = 4) [52, 58–60].

Plate designs could be pre-contoured by the manufacturers to the anatomical shape of the clavicle (*n* = 7) [50, 51, 54, 60, 61, 63, 65]. This design approach, also referred to as an anatomic design, is prevalent for locking plates. In contrast, some implants are modified during the surgery to better fit the shape of the clavicle, known as contoured implant (*n* = 8) [52, 53, 55, 57–59, 64, 66]. However, it is worth noting that the term “customized implant” was used to refer to both cases. While some studies presented in-house design, implant designs were majorly sourced from DePuy Synthes (West Chester, USA) [52, 54, 59, 62], its foundational company, Synthes GmbH (Oberdorf, Switzerland) [55, 58, 64], or its partnership company, AO Synthes (Solothurn, Switzerland) [56], in which there were later acquired by the Johnson & Johnson Medical Device (Irvine, USA). Other manufacturers from China included Aplus Biotechnology (Taiwan) [63], Beijing Naton Medical Group (Beijing) [66], Trauson Medical Instrument (Changzhou) [51, 53, 61]. One manufacturer was from the USA: Zimmer Biomet (Warsaw, USA) [66]. Some studies did not disclose their design source of implants but provided specifications, such as length, thickness, and screw configuration. Design modifications were also evaluated. These included the addition of a screw cap [56], a berried central hole [53, 57], a curved wing [57], grooving [53], and specific screws or wirings [59, 62, 65] (Fig. 4 and Table S1).

The positioning and design of the implant may have an impact on the stability of the fixation. As shown in Fig. 4, the most common placement in the reviewed articles was superior (*n* = 14) [50, 51, 54–64], followed by anterior placement (*n* = 5) [50, 54, 55, 58, 59]. Few studies also considered anterosuperior [59] and anteroinferior placement [59, 65]. Different placements have their own advantages, and the choice depends on the patient’s conditions and various other factors. Generally, anterior plate placement offers enhanced rigidity against bending, while superior plate fixation is more capable of withstanding axial compression and torsion [69]. Notably, anterior plating showed the highest chance of union but the lowest risk of revision surgery [70]. Furthermore, dual plating [54, 58, 65] and spiral plating approaches [53, 55, 63, 66] aimed to achieve radial fixation strength that strike a balance to ensure stability under multidirectional load [54, 63, 71]. It was reported that dual plating could reduce the rates of reoperation for symptomatic hardware removal [72].

## Contextual thematic analysis

### Modeling

Geometry reconstruction and mesh creation are two major steps before finite element analysis. Computed tomography (CT) scans have consistently been employed for model reconstruction across all studies, which serves as the conventional benchmark for evaluating bones and fractures. Most of the reviewed articles focused solely on reconstructing the clavicle; however, some extended to segment the trabecular core and cortical layers. One study expanded the scope to include other bones, including the scapula and humerus [50], and two studies considered soft tissues, such as ligaments, cartilage, and joint capsule [50, 54]. Given that nearly all clavicles were intact, fractures were simulated by introducing a gap. The details of the fracture gap distances are provided in Fig. 3 and Table A1. Although a postoperative patient was considered during the model reconstruction in one study, the timing and condition of the medical imaging remain unclear, so as the method the authors used to account for fracture reduction in the simulation [60].

**Table 1:** Material properties of bone and soft tissues in the review articles

Material	Component	Properties	Reference(s)
Cortical bone	Clavicle	*E* = 11,000 MPa, *v* = 0.3	[50]
		*E* = 17,000 MPa, *v* = 0.3	[53, 55–58, 61, 62, 64–66]
		Anisotropic *E* = 18,000 MPa (longitudinal) *E* = 8000 MPa (transverse), *v* = 0.3	[52, 59]
		Elastic modulus based on gray values of CT scans	[51]
	Scapula	*E* = 16,000 MPa, *v* = 0.3	[50]
	Humerus	*E* = 18,000 MPa, *v* = 0.3	[50]
Trabecular bone	Clavicle	*E* = 1000 MPa, *v* = 0.3	[53, 55–58, 61, 62, 64–66]
		*E* = 500 MPa, *v* = 0.1	[63]
	Fragment	*E* = 3 MPa, *v* = 0.4	[58]
Cartilage	Humerus cartilage	*E* = 0.66 MPa, *v* = 0.08	[50]
	Glenoid cartilage	*E* = 1.7 MPa, *v* = 0.08	[50]
	AC joint cartilage	*E* = 10.4 MPa, *v* = 0.3	[50]
Capsule	Ligament and capsule	*E* = 9.6 MPa, *v* = 0.3	[50]
	AC joint capsule	E = 24 MPa, v = 0.45	[54]

*AC* acromioclavicular, *E* Young’s (elastic) modulus, *v* Poisson’s ratio

In terms of the model reconstruction, Mimics (Materialise NV, Leuven, Belgium) is the most used software, while InVesalius (Centro de Tecnologia da Informacão Renato Archer, Campinas, Brazil) offers an open-source alternative for this application.

Less than half (*n* = 7) of the studies described the methods to reconstruct the model geometry of the implants. Figure 5 shows the different types of implant and surgical approaches. Most of them are modeled using computer-aided design software, including SolidWorks (Dassault Systèmes SolidWorks Corp., Waltham, USA) and Rhinoceros (Robert McNeel & Associates (TLM Inc.), Seattle, USA). Cheng et al. [51] proposed and modeled their own implant design, whereas other researchers employed a reverse engineering method, utilizing the specifications in product catalogs [50, 54, 61] or manual measurement [58] to inform their modeling. On the other hand, Cronskär et al. [52] and Marie [59] modeled the implants using a 3D scanner. All studies simplified the representation of screw threads as simple cylindrical shapes.

The reconstructed model geometry was optimized and assembled using various software programs, such as Magics (Materialise NV, Leuven, Belgium), 3-matic (Materialise NV, Leuven, Belgium), and Geomagic Design (3D Systems, Morrisville, USA). Subsequently, mesh generation was typically carried out using finite element software. However, some researchers opted for a separate mesh generation process prior to importing into the finite element software, utilizing tools such as Meshmixer (Autodesk, San Francisco, USA) and Hypermesh (Altair Engineering Inc., Troy, USA) for mesh creation and optimization. Two studies (*n* = 2) employed hexahedral elements to mesh some parts of their model assemblies [50, 52], but most of the studies favored tetrahedral elements, especially on the clavicle bone. While four studies (*n* = 4) did not specify the type of tetrahedron used [54–56, 60], seven studies (*n* = 7) implemented first-order linear tetrahedral (C3D4) [51, 53, 58, 61, 62, 65, 66], and the other six studies (*n* = 6) used second-order quadratic tetrahedral elements (C3D10) [50, 52, 57, 59, 63, 64].

The choice between C3D4 and C3D10 elements presents a trade-off between computational efficiency and accuracy. C3D4 elements are computationally less demanding and easier to generate, making them suitable for complex geometries and large models [73]. However, they are prone to shear and volumetric locking, particularly in bending-dominated problems. In contrast, C3D10 elements offer superior accuracy and are less susceptible to locking phenomena, providing more reliable results with fewer elements [73]. This improved performance comes at the cost of significantly higher computational demands and increased difficulty in initial mesh generation.

The finite element analysis software utilized across the studies included Abaqus (Dassault Systèmes SIMULIA, Vélizy-Villacoublay, France), Ansys (Ansys Inc., Canonsburg, USA), and MSC Marc (Hexagon MSC Software Corp., Newport Beach, USA), with their usage reported in ten (*n* = 10), four (*n* = 4), and one (*n* = 1) studies, respectively. Additionally, two studies did not disclose the specific software employed for their analysis.

### Materials

The material properties for tissues and implants are shown in Tables 1 and 2, respectively. The clavicle bone is often segmented into the cortical layer and trabecular core and there is a general consensus regarding their material properties. The elastic moduli (or Young’s modulus) for the cortical and trabecular bone of the clavicle are 17,000 MPa [53, 55–58, 61, 62, 64–66] and 1000 MPa [53, 55–58, 61, 62, 64–66], respectively. Both exhibit the same Poisson’s ratio of 0.3. Some studies have explored a more complex material model. For example, anisotropic properties with different stiffnesses along the transverse and longitudinal directions [52, 59] and non-homogeneous properties based on the gray scale (Hounsfield value) of the CT scans [51]. The constitutive equation for the non-homogeneous properties was adapted from another source [74]. However, the authors of the reviewed article did not specify which set of regression equations and coefficients were used [51]. Equations 7–10 were extracted from part of the results from Rho et al. [74] that involved the test results of proximal humerus fitted with linear regression.

7 $${E}_{SI}=-270+4.25\rho$$

8 $${E}_{ML}=-201+2.50\rho$$

9 $${E}_{AP}=-169+2.22\rho$$

10 $$\rho =173+0.624{\Delta }_{HU}$$

where *E* represents the elastic modulus (expressed in MPa) in superior-inferior (*SI*), medial–lateral (*ML*), and anterior–posterior (*AP*) directions (in subscript). *ρ* and △_*HU*_ denote the apparent density in kg/m^3^ and Hounsfield value in CT, respectively.

**Table 2:** Material properties of implant in the review articles

Material	Properties	Reference(s)
Titanium alloy	*E* = 110,000 MPa, *v* = 0.3	[50, 51, 55, 63]
	*E* = 110,000 MPa, *v* = 0.33	[64]
	*E* = 110,000 MPa, *v* = 0.34	[54]
	*E* = 114,000 MPa	[56]
	*E* = 186,400 MPa, *v* = 0.3	[53, 57, 59, 65, 66]
Stainless steel	*E* = 186,400 MPa, *v* = 0.3	[52, 61, 62]
	*E* = 193,000 MPa, *v* = 0.3	[51]
	*E* = 193,000 MPa, *v* = 0.31	[57]
	*E* = 200,000 MPa, *v* = 0.3	[58]
Maraging steel	*E* = 130,000 MPa, *v* = 0.3	[57]
Magnesium alloy	*E* = 44,000 MPa, *v* = 0.27	[51]

*E* Young’s (elastic) modulus, *v* Poisson’s ratio

Furthermore, Kritsaneephaiboon et al. [58] assigned relatively low stiffness to the bone fragment, with an elastic modulus of 3 MPa and a Poisson’s ratio of 0.4. Calişal and Uğur [50] expanded the model to encompass the shoulder complex, incorporating the material properties of the scapula, humerus, cartilage, and capsule. They highlighted that they have assigned viscoelastic material properties to the soft tissue but relevant information was missing [50]. Moreover, Huang et al. [54] considered the material properties of the acromioclavicular joint capsule.

Existing papers have evaluated titanium alloy, stainless steel, maraging steel, and magnesium alloy as materials for use in implants (Table 2). Regarding titanium alloy, there were generally two schools of thought: one assigned a stiffness of 110,000 MPa [50, 51, 54, 55, 63, 64], while the other opted for 186,400 MPa [53, 57, 59, 65, 66]. However, it is important to note that the material properties for both titanium alloy and stainless steel overlapped at 186,400 MPa [52, 61, 62]. The source and justification for these values should be carefully considered. Notably, stainless steel exhibits the highest elastic modulus at 200,000 MPa [58], whereas that of magnesium alloy appears to be the lowest, standing at 44,000 MPa [51].

### Loading scheme

**Fig. 6:**
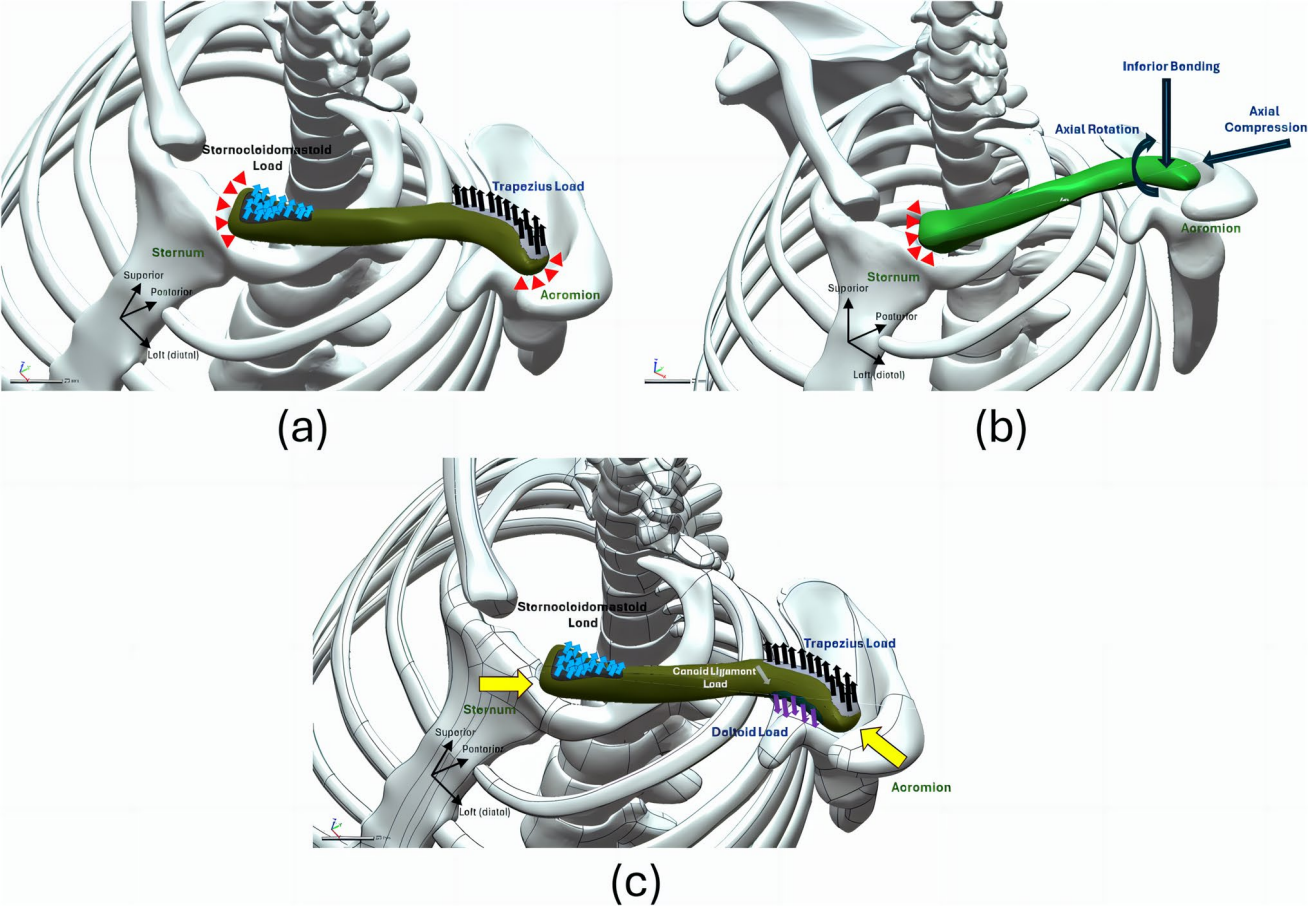
Illustration of boundary and loading conditions of the finite element analysis for: **a** axial rotation, inferior bending, and axial compression, respectively; simulating coffee-drinking by **b** sternocleidomastoid and trapezius load with proximal and distal end fixed and **c** with an addition of proximal and distal joint force, as well as deltoid and conoid ligament load

Among the reviewed articles, the most tested loading conditions were pure loading conditions (Fig. 6 and Table A1), which included axial rotation, axial compression, and inferior bending. Besides, Han et al. [53] attempted to simulate a combined loading condition of the pure loadings. To apply the boundary and loading conditions, at the proximal end near the sternum, constraints were applied, and the loads were exerted at the distal acromion end (Fig. 6a). Specifically, a force of 100 N was used for bending and compression, while a torque of 1 Nm was applied for axial rotation. Some variations were observed at 200 N [63], 250 N [64], and 4 Nm [63]. However, these values appear to be arbitrary.

Another common set of simulation scenarios involved modeling the drinking motion. These studies typically originated from the same research team or network [52, 54, 59], despite the actual boundary and loading conditions applied were slightly different (Fig. 6). Specifically, they employed a multibody musculoskeletal model (AnyBody Modeling System, AnyBody Technology, Aalborg, Denmark) to simulate the static position of holding a 0.5 kg cup of tea in front of the mouth. The muscle forces and joint forces were then utilized as input for the boundary and loading conditions in the finite element analysis. Huang et al. [54] constrained the proximal end of the clavicle and the acromion, which were connected via a capsule. They then applied a distributed force to the attachment sites, simulating the forces exerted by the sternocleidomastoid and trapezius muscles. Besides, the other two studies replaced these constraints with contact forces at the sternoclavicular and acromioclavicular joints [52, 59]. Additionally, they considered the deltoid muscle and the conoid ligament forces. Two other studies mentioned that they simulated shoulder motion (abduction and flexion) and applied muscle forces (deltoid and pectoralis anterior), respectively [50, 60]. However, the exact procedures, such as the specific sites for applying and defining constraints, loads, and motions remained unclear.

Interaction properties between the components also play a critical role in defining boundary condition. To emulate the behavior of locking screws without explicitly modeling the threads, the bone-to-screw and screw-to-plate interfaces were assumed to be tied or coupled [51–56, 58, 59, 61–65], which allowed efficient simulations while capturing essential contact characteristics. On the other hand, there were variations in how researchers defined the interaction between the bone and the plate. Some studies simplified the interaction as frictionless [53–55, 63, 66]. Other studies considered the coefficient of friction with values, such as 0.3 [51, 58] or 0.42 [62]. Furthermore, bone-to-bone contact or interfragmentary contact became crucial when fragments were expected to touch each other, especially in cases of fixation of comminuted fractures. They might be assumed frictionless [61] or with a coefficient of friction, such as 0.2 [63, 66] or 0.46 [62].

### Model validation and verification

**Fig. 7:**
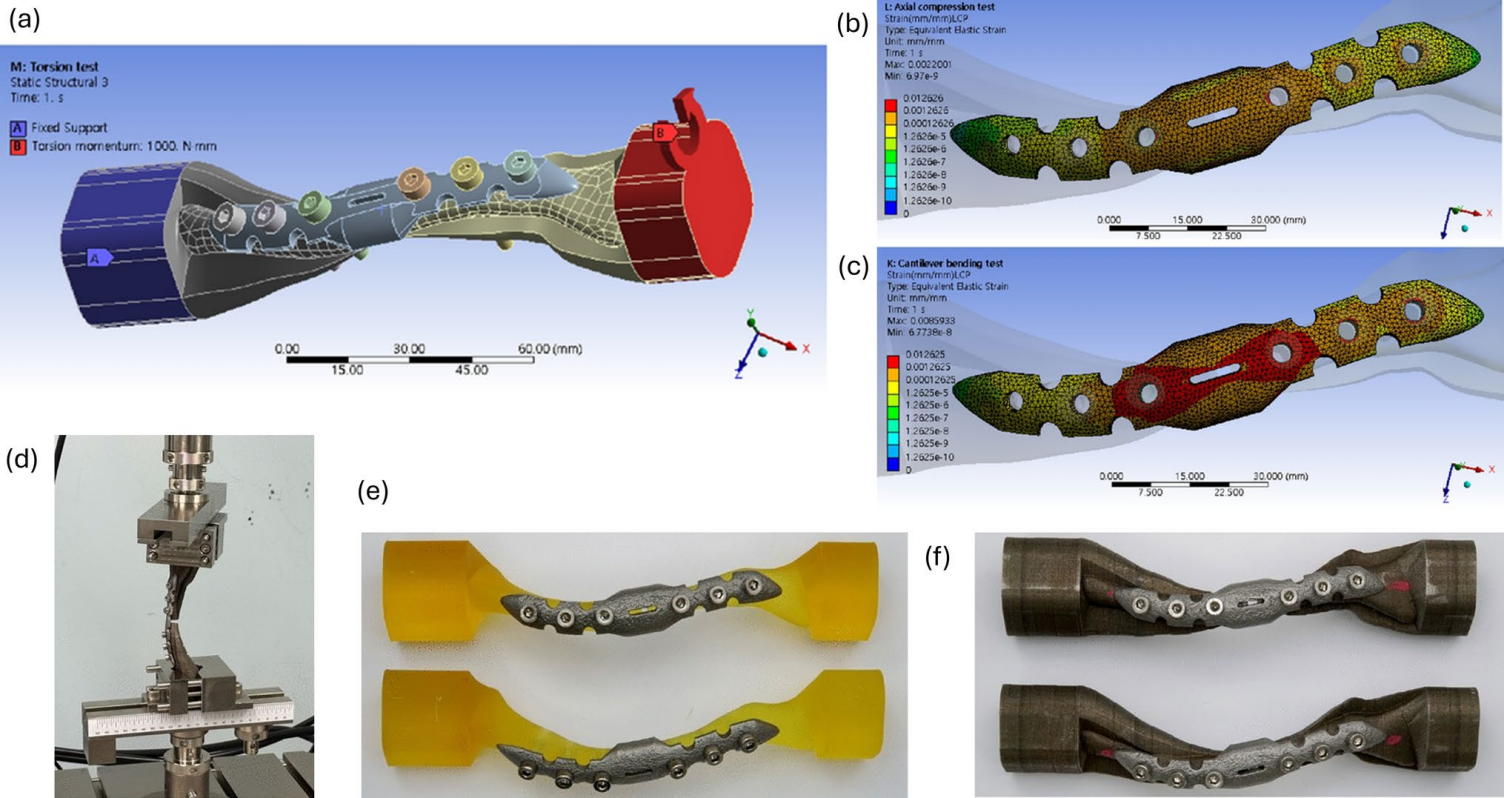
An excerpted illustration of the model validation process conducted by Kim et al. [57]: **a** finite element model resembling the physical testing process; **b** finite element simulation results of axial compression; **c** finite element simulation results for inferior bending; **d** setting of mechanical testing of specimen; **e** 3D printed resin clavicle fixed with implant for model validation; **f** 3D printed metal clavicle fixed with implant for model validation (figures reproduced under Creative Commons Attribution License)

A limited number of studies have undertaken model validation. Among 17 studies reviewed, only eight (*n* = 8) underscored model validation, and merely two (*n* = 2) carried out direct model validation within their studies [56, 57]. Specifically, Kim et al. [56] estimated the changes in average stiffness of a locking plate with and without a screw cap, albeit not considering the bone model, compared to simulation results. The same team, in a subsequent paper [57], conducted mechanical testing on locking plate affixed to 3D-printed metal and resin models of clavicles. Then, they replicated the mechanical testing process by constructing jigs at the ends of the clavicle models and applied the same load for the simulation (Fig. 7). The remaining studies (*n* = 6) performed indirect model validation by juxtaposing their results with those from existing studies [51, 53, 61, 64–66].

While the finite element calculations from the commercially available software package have been benchmarked, further model verification can be achieved through a mesh convergence test, mesh quality assessment, and sensitivity analysis. Nine studies (*n* = 9) conducted model verification, with six (*n* = 6) focusing on mesh convergence test, two (*n* = 2) on mesh quality assessment, and one (*n* = 1) on sensitivity analysis. Although some studies lacked a clear process and results for the mesh convergence test, the recommended convergence criteria are either 2% or 5% [51, 54, 62, 63]. Huang et al. [54] aimed for a converged solution regarding fragment displacement in one of the implant designs. In contrast, Kritsaneephaiboon et al. [58] focused on achieving convergence on the maximum von Mises stress across all implants under axial compression. Mesh quality assessment was concerned on orthogonal quality and skewness [57]. Cronskär et al. [52] followed the recommendation of the software that the element angle should be less than 25° and the edge length no more than 2 mm. On the other hand, Marie [59] conducted a sensitivity analysis to quantify and justify the minimal variation in plate displacement with various bone material properties.

## Conceptual thematic analysis

### Independent and dependent factors

All studies, with one exception, pinpointed the implant design or approach as their primary independent variables (Table S3). Several studies (*n* = 11) also incorporated a secondary independent variable, the loading case, to evaluate the impact of the implant design or approach under various loading conditions. Five studies (*n* = 5) considered implant designs and configurations [54, 55, 58, 63, 65], particularly the spiral plate [55, 63, 65] and dual plate [54, 58, 66]. Furthermore, three studies (*n* = 3) made comparisons between the plating and intramedullary approaches [61, 64, 66]. Design modifications were also taken into account, including changes to the plate shape [53, 57], berried holes [53, 57], and variations in screw/wiring selections and configurations [59, 62, 65]. On the other hand, three studies (*n* = 3) investigated the placement of the implant [50, 59, 66], and one study examined the impact of implant materials [51].

Von Mises stress of the bone and/or implant was consistently reported in all the reviewed articles. Von Mises stress, also referred to as the maximum distortion energy or equivalent stress, is a comprehensive measure that accounts for stress in all loading modes, including tension, compression, and shear, particularly applicable to ductile materials like metals. Its comprehensive and convenient nature, eliminating the need for separate calculations of different types of stress, contributes to its widespread usage. Mathematically, von Mises stress, *σ*_vms_ could be calculated by the principal stresses (Eq. 11).

11 $${\sigma }_{\text{vms}}=\sqrt{\frac{1}{2}[{\left({\sigma }_{1}-{\sigma }_{2}\right)}^{2}+{\left({\sigma }_{2}-{\sigma }_{3}\right)}^{2}+{\left({\sigma }_{1}-{\sigma }_{3}\right)}^{2}]}$$

where *σ*_*1*_, *σ*_*2*_, and *σ*_*3*_ are the maximum (tensile), intermediate, and minimum (compressive) principal stress.

The von Mises stress criterion, or the maximum distortion energy theory, posits that the yielding of material starts when the von Mises stress, which represents the elastic energy of distortion, reaches the yield strength. Within the context of this review, high levels of von Mises stress in the implant could suggest a potential risk of implant failure, whereas a high von Mises stress in the bone might hinder the healing process and bone union.

Promoting the stability of the fracture site to facilitate union has been one of the focuses of many studies. Most loading schemes fixed one end of the clavicle and allowed the other end to move. In this manner, the construct stiffness (or rigidity) for a specific loading mode is determined by the ratio of the applied load to the maximum displacement of the movable end [51, 54, 59, 61, 63–66]. Cheng et al. [51] and Kritsaneephaiboon et al. [58] conducted a more localized investigation into the strain of the bone at the fracture site, where a higher elastic strain indicates more deformation, and consequently, less fixation stability. Additionally, the micro-motion of the fracture gap was computed by the changes of the averaged gap distance between the nodes of the gap under load-bearing conditions [61, 62, 65, 66]. Meanwhile, there is currently no consensus on the parameter to quantify the stability of the fracture site.

### Optimal fixation methods for different fracture types

Finite element analyses have provided valuable insights into the biomechanical performance of various fixation methods for different clavicle fracture types. For midshaft transverse fractures, which constituted the majority of cases, locking plates have demonstrated superior stability. Zeng et al. [64] found that the locking plate exhibited higher construct stiffness than the nailing approach (using titanium elastic nail), indicating enhanced stability. Additionally, the nails led to increased stress in both the bone and implant, particularly around the fracture site, potentially disrupting bone union and leading to implant failure. Similarly, Ni et al. [61] found that locking plates had approximately 60% higher bending construct stiffness and 30% higher compressive construct stiffness compared to intramedullary nails (Sonoma intramedullary nail and Rockwood clavicle pin). However, their biomechanical responses to bending and compression varied. Locking plates exhibited higher implant stress and lower bone stress than nailing during bending, and the opposite during compressive loading. Notably, the stress of the nail approached its yielding limit. Furthermore, Zhang et al. [66] showed that the Herbert screw fixation did not provide better structure stiffness and stability against bending compared to plating. These biomechanical findings, which highlight the poor stability and risk of implant failure in the nailing approach, align with the clinical observations [75, 76].

For comminuted fractures, dual plating techniques have shown lower implant stress and bone strain at the fracture site under bending loads, while performing comparably to single plating under torsional loads [58]. In a separate study, Ni et al. [62] evaluated the augmented fixation on locking plates using cerclage wirings and interfragmentary screws. They recommended the use of double interfragmentary screws to achieve lower bone stress and higher stability. However, a direct comparison between dual plates and augmented locking plate fixation with double interfragmentary screws is currently lacking.

These findings suggested that locking plates are recommended as the primary fixation method for simple midshaft fracture due to their superior stability and higher construct stiffness. Intramedullary nails should be used with caution, considering their higher biomechanical risk of implant failure and disruption of bone union. Dual plating techniques are recommended for comminuted fractures, while augmented fixation using locking plates with double interfragmentary screws is a viable alternative.

### Plate positioning and its impact on fixation stability

Different implant placements may result in a construct that is robust against various directions of forces. Huang et al. [55] and Marie [59] demonstrated that anterior placement could result in reduced peak stress, compared to that of superior placement, even though Marie [59] might actually favor superoanterior placement that might be more effective in lowering the bone stress levels. In addition, Calişal and Uğur [50] discovered that superior placement resulted in fourfold and 1.6-fold higher bone stress during shoulder abduction and flexion, respectively, compared to anterior placement, suggesting a risk of bone nonunion. They also showed that superior placement remarkably increased stress on the plate, screws, acromioclavicular ligament, and the glenohumeral joint. Despite the strength of anterior plating in structural stiffness against bending and torsion, it shall be noted that it might not be very effective against axial compression and might also impose high stress on screws during bending [55].

Meanwhile, spiral and dual plating strategies were also employed in an attempt to enhance stability beyond that of traditional locking plate or single plating. However, these approaches seemed to rank in the middle compared to other techniques [55]. Spiral plating exhibited inferior performance in structural stiffness in all loading modes to anterior placement. It induced a von Mises stress that is 260 MPa higher than that of anterior placement, slightly less stress during torsion, and stress comparable to that of anterior placement during compression [63]. On the other hand, the advantage of the dual plating strategy was its stability and resilience against bending. Huang et al. [54] suggested that dual plating presented the least displacement, the lowest implant stress, but the highest bone stress compared to the hook plating and superior placement of a single plate. Kritsaneephaiboon et al. [58] demonstrated that dual plating resulted in lower implant stress and elastic strain at the bone fracture site. However, it also led to high bone stress, which was comparable to that of superior placement.

Current biomechanical simulation studies suggested that anterior plating remains a versatile option. This aligns with clinical observations indicating that superior plating might be associated with a higher rate of symptomatic hardware [77]. For complex fractures or oblique fractures where multidirectional stability is crucial, spiral or dual plating techniques may offer additional benefits. However, while anteroinferior plating is often highlighted in the literature [69], there is a notable lack of study directly comparing it to other approaches in our review, which makes it challenging to draw conclusions about the optimal plating position.

### Implant design considerations for improved outcomes

Implant design and configuration modifications in the reviewed studies often aim to address the issue of stress concentration and localized implant failure. Given that stress frequently concentrates near fracture sites, various approaches were explored to alleviate this problem. Finite element analyses have supported the effectiveness of several strategies in redistributing localized stress and lowering the stress levels of the implant around the fracture zone. These strategies included incorporating screw caps, utilizing berried holes, and implementing fillet or filleting techniques at stress concentration sites [53, 56, 57]. The selection of screws also plays a crucial role in stress distribution. For instance, the use of lag screws has been shown to distribute stress more evenly across the plate, effectively reduce the high stress concentration at implant [59]. Furthermore, specific design modifications, such as eliminating notches and filling empty screw holes, can significantly reduce the overall implant stress. Under axial compression, these modifications have been observed to decrease stress by approximately 240 MPa [53].

Precontoured or anatomically shaped implants were believed to offer superior fit and stability compared to conventional straight plates in clavicle fracture fixation. The rationale behind this assumption was that these implants are designed to match the complex three-dimensional anatomy of the clavicle more closely, potentially leading to better fracture reduction and stability. In our review, one study by [61] compared an anatomically shaped interfragmentary nail to a straight pin, demonstrating that the former induced less fracture micro-motion at the fracture site and less bending stress on the implant. These findings suggested that anatomically shaped implants may provide biomechanical advantages over their straight counterparts. Kim et al. [57] also proposed a double-curved wing design to enhance the radial fit of the implant and found to reduce stress concentration at the fracture area. However, it is important to note that current evidence lacks direct comparative studies between precontoured or anatomically shaped plating and straight plates for clavicle fractures.

In summary, implant modifications, such as hole settings and screws, could reduce the risk of implant failure by redistributing stress and lowering the peak stress level of the implant. Anatomically shaped implants might provide better biomechanical conditions at the fracture site to reduce the chance of non-union and delayed healing. However, there is a notable lack of comparative studies between shaped and non-shaped research.

## Discussion

### Critical role of biomechanical insights in enhancing of clavicle fracture surgery

The clavicle, characterized by its distinctive double-curved S-shaped geometry, plays a crucial role in connecting the axial skeleton to the shoulder girdle [78]. This unique bone not only serves as an anchor for the movements of the glenohumeral, acromioclavicular, and scapulothoracic joints but also acts as a vital protector for underlying organs and neurovascular structures [78]. The clavicle’s complex geometry, coupled with its biomechanical properties and the multifaceted loading modes it experiences, might make it vulnerable to traumatic injuries. Midshaft fractures are common, often resulting from falls on an outstretched hand or direct blows, frequently seen in contact sports and road traffic accidents [79]. Furthermore, these factors present significant challenges in determining optimal implant design and surgical approaches for fracture management. Complications such as non-union and implant failures are not uncommon in clavicle fracture fixation [16, 19, 78], with an overall complication rate of 17% [80]. In this context, the application of numerical models and finite element analyses has become invaluable for research into clavicle fracture fixation. They provide insights into internal stress and strain distribution under various fracture scenarios, fixation strategies, and loading conditions, which are often difficult or impossible to assess through traditional experimental methods. It facilitates non-invasive quantitative assessment with prediction that might be more accurate than measurement alone [81]. This approach not only deepens our understanding of the biomechanical implications of different fixation techniques but also paves the way for personalized and precision medicine in fracture management [82].

### Comparative analyses and clinical relevance

Our analyses suggested a preference for locking plates in terms of fixation stability. This aligns with some clinical observations, particularly regarding early recovery rates. Clinical studies have shown that plate fixation leads to faster early recovery than nailing, especially in comminuted fractures [83] [84]. However, it is important to note that intramedullary nailing has demonstrated advantages in terms of lower infection rates and fewer implant failures [68, 84]. Two meta-analyses comparing plate fixation and intramedullary fixation provide further nuance to these findings [68, 85]. One study reported no significant difference between the two methods in terms of long-term function and non-union rates for non-comminuted, displaced midshaft clavicle fractures [85]. However, it noted a higher rate of re-fracture after implant removal with plate fixation. Another meta-analysis reached similar conclusions regarding long-term function but suggested that plating might carry a higher risk of non-operative complications [68]. It is crucial to emphasize that our findings primarily focus on biomechanics, specifically fixation stability and implant stress. While these generally align with clinical results for early recovery and union, they may fail to account for challenges in surgical operations and non-operative complications. The issue of re-fracture after implant removal, highlighted in clinical studies, is an interesting topic that warrants further investigation through finite element analysis.

Regarding plate positioning, our review suggested a preference for anterior plating from a biomechanical perspective. This aligns with some clinical observations indicating that superior plating might be associated with a higher rate of symptomatic hardware than anterior plating [77]. A meta-analysis comparing anterior-inferior plating and superior plating showed that anterior-inferior plating was superior in reducing union time, operation time, and blood loss [69]. However, another clinical study found no significant difference between superior and anterior plating regarding implant removal, healing, complications, and functions [86]. Besides, our review also indicated a preference for precontoured and/or anatomically shaped implants or better-fitted designs from a biomechanical standpoint. This is supported by clinical evidence, with one study reporting that precontoured plates were associated with lower rates of hardware removal [87] and another showing higher union rates with precontoured plates [18].

Our review revealed a notable gap in the literature: no studies directly compared comminuted and non-comminuted fractures, limiting our ability to draw conclusions about the relative effectiveness of different fixation methods for these specific fracture types. Similarly, we found no comprehensive studies that tested all common plating options and placements within a single model, which constrains our ability to make generalized recommendations. This limitation is not unique to finite element studies; clinical trials often face similar challenges in comprehensively comparing all plating options due to practical and ethical constraints. However, finite element studies have the potential to address these limitations by developing a set of model databases that can simulate various conditions, including different implant options and placements within the same model. Future research should focus on bridging the gap between finite element analysis and clinical outcomes, potentially leading to more robust and comprehensive guidance for clavicle fracture fixation.

### Limitations of finite element studies in clavicle fracture fixation

A significant limitation in the reviewed finite element studies was the prevalent use of single-subject and subject-specific models. This approach, while valuable for patient-specific analysis, raised concerns about the generalizability of the findings. Such a study design approach may fail to capture the variability in bone geometry and mechanical properties that exist among individuals. Furthermore, the majority of studies did not utilize models from actual fracture cases. Instead, they reconstructed clavicle models from intact bones and simulated fractures by creating artificial gaps. This method might fail to replicate the true and natural complexity of fracture patterns, including irregularities, non-plantar surfaces, and variations in fracture sites [88]. These factors could significantly influence load transfer in the simulation, potentially impacting the implications for bone union. Additionally, the dimensions of these manually created fracture gaps often appear arbitrary. In some cases, gaps as large as 10 mm were simulated, which did not reflect clinical reality where one graft would typically be employed for such large defects [89]. This discrepancy between simulation and actual surgical practice further limited the clinical applicability of the findings.

Another limitation in the majority of reviewed studies was the focus on modeling the clavicle bone in isolation, rather than considering the entire shoulder girdle complex. This approach omitted crucial components such as joint capsules, cartilage, and muscles, which play essential roles in load distribution and biomechanical behavior. The absence of these structures, particularly the sternoclavicular and acromioclavicular joints, may lead to an incomplete representation of the biomechanical environment. Only two studies included soft tissues, such as ligaments, cartilage, and joint capsules in their models. While some studies attempted to represent muscular action through applied forces or distributed loads, this approach may not fully capture the complex, dynamic nature of muscle (belly) and bone interactions. Regarding material properties, there has been some progress in implementing heterogeneous bone properties based on density distributions. The incorporation of more sophisticated material models, such as hyperelastic or viscoelastic properties, particularly for soft tissues, could enhance the physiological relevance of these simulations to better account for non-linear and time-dependent responses of tissues, potentially leading to more accurate predictions.

The use of simplified loading schemes may not accurately represent the complex biomechanical environment experienced by the clavicle during daily activities. Most studies applied pure loading modes, such as axial compression, bending, or torsion. While these provided valuable baseline data for comparison with cadaveric experiment, they fell short for capturing the multidirectional and dynamic loads experienced during functional activities. Determining appropriate physiological loading conditions for simulations presents a significant challenge. Real-world clavicle loading involves complex interactions of muscle forces, joint reactions, and external loads that vary with arm position and movement. To address this limitation, some researchers have incorporated more sophisticated approaches to estimate loading conditions. For example, motion capture systems or inertial measurement units combined with musculoskeletal modeling and/or electromyography have been employed to estimate more complex loading scenarios that include muscle forces [90, 91]. In our review, we identified studies that utilized this approach to simulate coffee drinking motion, providing a more realistic representation of daily activities. However, this represents a research gap for more research into critical loading schemes that occur during daily activities and may be crucial for fracture union.

The selection of outcome measures in previous studies has centered on the premise that lower bone stress is preferable to avoid bone failure (or yield). However, this assumption is challenged by the concept of stress shielding, as pointed out by one of the papers, suggesting that other parameters, such as interfragmentary compression [92] and strain energy density [93], may be more relevant to addressing the non-union and bone remodeling issues. In addition, the compression of fragments is likely more indicative of stability and union than the simplistic gap model. Von Mises stress might not take into account different loading modes separately. Different kinds of stress fields, including tension, compression, and shear under various loading modes, especially along the trabecular core and cortical layer [94], could provide more comprehensive information on how the implant could be designed or modified to alleviate stress concentrations. Parametric analysis can be conducted to assess the impact of various design parameters and features [95].

### Study quality and model validation

The methodological quality assessment conducted using MQFESS revealed significant areas for improvement across the reviewed studies. With an average score of approximately 50%, it is evident that there was substantial room for enhancement in the overall quality of these modeling studies, particularly in justifying unplanned analyses and presenting comparative plots with consistency and clarity.

A more comprehensive discussion on model assumptions and validity is critically needed. While all studies addressed the implications of their findings, and over half discussed limitations related to loading schemes and external validity issues, many failed to adequately address limitations associated with modeling techniques and materials, as well as internal validity concerns and uncertainties inherent in the modeling process. An explicit discussion of the modeling process, including geometry reconstruction, part inclusion, material properties, and loading schemes, is essential for ensuring transparency and reproducibility [44]. Furthermore, a contextual evaluation of how well the model and loading cases align with common real-world scenarios, coupled with a sensitivity analysis demonstrating how changes in model settings or assumptions affect outcomes, would better quantify external validity, internal validity, and uncertainty of the simulation findings [44, 96]. This approach would also enhance the clinical relevance of the results.

Model validation emerged as a particularly significant area of concern, with the relevant MQFESS domain receiving a score of only about one-third. This low score reflected substantial shortcomings in model validation and verification processes. Many studies either did not undertake these processes or completed only one of the two. Some merely stated that they had completed validation or verification without presenting any results. This lack of validation and verification raised serious questions about the reliability and accuracy of the presented models, even before considering the quality of these processes. Some researchers commented on the inherent insufficiency of model validation or verification processes, given the paradox of needing simulation if physical experiments could be arranged [44]. While indirect measurements have often been used as validation metric to address this issue, providing multiple levels or diverse measurement data could improve model credibility [44].

### Proposed solutions and future directions

The prevalence of single-subject and subject-specific models in clavicle fracture fixation studies can be attributed to the substantial time and effort required to create even a single finite element model [44]. However, this approach limited the generalizability of findings and may not capture the full range of anatomical variations present in the population. To address these limitations and enhance the efficiency and applicability of finite element modeling in this field, several promising avenues for future research emerge. One such approach is the use of statistical 3D models. Statistical shape modeling utilizes large datasets of anatomical structures to create a mean shape and capture the primary modes of shape variation within a population [97]. This method could enable the generation of representative clavicle models that encompass a wider range of anatomical variations, potentially improving the external validity of finite element studies. By incorporating statistical shape models, researchers could more efficiently explore the impact of anatomical variations on fracture fixation outcomes without the need to create individual models for each variation.

Another promising direction is the application of AI-driven methods to automate and accelerate various aspects of the model reconstruction process [98]. Deep learning techniques have shown significant potential in automating the segmentation of bone structures from medical images [99], optimizing mesh quality and density [100], and even directly estimating finite element predictions [101]. Furthermore, future studies could benefit from a more comprehensive approach to accounting for variations in fracture patterns. Some researchers have utilized fracture maps to quantify and visualize probability distributions of fracture lines from population data [102, 103]. This approach could be integrated into finite element studies of clavicle fractures, allowing for repeated simulations using Monte Carlo methods based on the fracture map data. Such an approach would improve the representation of variability in fracture patterns, potentially leading to more clinically relevant and generalizable results.

Building upon the identified limitations in existing loading schemes, a more physiologically relevant approach is proposed for future studies. Unlike the lower limb, where gait analysis provides a well-established and representative loading scenario [36, 37], the upper limb presents unique challenges in determining a standardized, functionally relevant loading scheme. To address this, we propose simulating specific maneuvers that may pose a high load to the clavicle or shoulder girdle complex that potentially led to non-union, which includes reaching, lifting a heavy load, and push-up position (simulating getting up from bed). Boundary and loading conditions for finite element simulations can be derived from various motion capture methods, including optical systems, inertial measurement units, or image-based techniques. These systems measure kinematics, which can then be input into musculoskeletal models to estimate joint contact loads and muscle forces.

One of the critical challenges in finite element analysis of clavicle fracture fixation is the validation of computational models. A particularly innovative approach, as demonstrated in one of the reviewed studies, involved using scaled model validation with 3D-printed replicas. This method offers several advantages for improving the reliability and clinical relevance of finite element models. The process begins by 3D printing the bone based on the computational models, creating tangible replicas that closely mimic the geometry of the original clavicle. These printed models then undergo various mechanical tests under different loading conditions using standardized mechanical testing machines. The material properties of the 3D-printed model and the applied loading conditions are then fed back into the simulations, allowing for a direct comparison between physical test results and computational predictions. To further strengthen this approach, future studies could incorporate sensitivity analyses by varying material properties through the use of different 3D printing materials/densities and exploring a range of loading conditions. However, it is crucial to acknowledge the limitations of this validation approach. The validation is primarily confined to biomechanical (physics) aspects [104] and cannot account for biological factors such as bone healing, vascularization, tissue response to implant, or long-term effects. Therefore, to ensure clinical relevance and applicability, it is imperative to complement these biomechanical simulations with rigorous clinical validation studies [105].

### Limitations of this review

This review has several limitations. Firstly, our search was restricted to English language sources, which may have introduced language and selection bias. Secondly, we limited our search to prominent databases and specific publication types, such as journal articles and full conference papers, potentially overlooking relevant studies in other formats or less mainstream sources.

A significant limitation was the small number of studies included, and the limited number of clavicles simulated (*n* = 22) across these studies. This small sample size, coupled with the heterogeneous nature of the studies and models not extracted from representative cases, necessitated caution in interpreting and generalizing the biomechanical performance findings. The diversity in modeling techniques, fracture types, and implant designs across this limited sample may not fully represent the wide range of clinical scenarios in practices. To maintain homogeneity and credibility, we excluded studies that did not reconstruct clavicle models from participants’ medical images. While this decision enhanced the review’s internal validity, it may have led to the exclusion of valuable data. Some studies using models reconstructed from cadavers or “representative” norm geometry from purchased sawbones or 3D anatomical atlases [106, 107] could offer insights, albeit with limitations in validity and credibility.

Future reviews might consider conducting a meta-analysis of single-subject designs to integrate evidence from subject-specific models into a case series collection. This approach could provide a higher level of evidence comparing different implant designs and surgical approaches. Additionally, future studies should prioritize the use of patient-specific models (instead of surrogate intact models), to enhance the clinical relevance of finite element analyses in clavicle fracture fixation research.

## Concluding remarks

This systematic scoping review consolidates the evidence and methodologies in 3D numerical modeling and finite element analysis applied to the clavicle fracture fixations. The process involved the reconstruction of an intact clavicle using CT scans, followed by the creation of a gap to simulate a fracture. On the other hand, the modeling of implant was accomplished through reverse engineering by means of manual measurements, specifications from product catalogs, or 3D scanning. While the material properties were often adopted from existing literature, pure loading schemes, including axial compression, inferior (cantilever) bending, and axial torsion, are common boundary and loading conditions. Some studies attempted to estimate the muscle loadings on the clavicle of daily tasks (such as drinking coffee) using musculoskeletal modeling. Model validation can be carried out by replicating a mechanical test on a 3D-printed specimen, while model verification can be performed through a mesh convergence test and mesh quality assessment.

The primary focus of the review articles lies on the comparison of different implant designs, configurations, and placements under different loading conditions on the risk of failure and the stability of the fracture site. The risk of failure was commonly evaluated by the von Mises stress of the bone and implant, whereas the stability was assessed by construct stiffness, strain, and micromotion. In general, the review articles seem to favor the anterior plating approach, and they recommended design modifications to alleviate the stress concentration of the implant near the fracture site. Given the complex loading conditions and the fact that different implant configurations are suited to different loading modes, it is imperative to identify the most common loading case in real-life and consider variations in the characteristics of the fracture. This approach will provide a comprehensive picture of which configurations are suitable for specific scenarios.

Our review findings suggested a preference for plate fixation, particularly with anterior placement, for midshaft transverse fractures. However, it is important to note that due to the limited number of studies focusing on specific fracture types, it is challenging to provide definitive recommendations for all fracture scenarios. To address this limitation, we recommend the development of a comprehensive database of finite element models. Such a database would allow researchers to test various implant options and placements on the same set of models, potentially leading to more robust and generalizable recommendations. Furthermore, clinical validation of these finite element studies is crucial to ensure that the biomechanical findings translate effectively to real-world patient outcomes.

## Supplementary Information

Below is the link to the electronic supplementary material.

Supplementary file1 (DOCX 45.7 KB)
