# Supplementary material for: Finite element modeling of clavicle fracture fixations: a systematic scoping review
Source: Med Biol Eng Comput. 2025 Jan 28;63(6):1585–607. doi: 10.1007/s11517-025-03294-1 (PMC12106152; doi:10.1007/s11517-025-03294-1)
Supplement: Supplementary file 1 — Supplementary file1 (DOCX 45.7 KB) [file 11517_2025_3294_MOESM1_ESM.docx]

**Table S1 Raw results of Methodological Quality Assessment using MQFESS**

| Study | Calişal and Uğur (2018) | Cheng, et al (2021) | Cron skär, et al. (2015) | Han, et al (2023) | Huang, et al. (2016) | Huang, et al. (2023) | Kim, et al. (2022) | Kim, et al. (2024) | Kristane ephaiboon, et al. (2024) | Marie (2015) | Marinescu, et al (2017) | Ni, et al. (2016) | Ni, et al. (2020) | Wu, et al. (2021) | Zeng, et al. (2015) | Zhang, et al. (2019) | Zhang, et al. (2020) | % |
| --- | --- | --- | --- | --- | --- | --- | --- | --- | --- | --- | --- | --- | --- | --- | --- | --- | --- | --- |
| Q1 | 1 | 1 | 1 | 1 | 1 | 1 | 1 | 1 | 0 | 1 | 0 | 1 | 1 | 1 | 1 | 1 | 1 | 88% |
| Q2 | 1 | 1 | 0 | 1 | 1 | 1 | 1 | 0 | 1 | 0 | 0 | 0 | 0 | 1 | 0 | 0 | 0 | 47% |
| Q3 | 1 | 1 | 0 | 1 | 1 | 1 | 1 | 1 | 0 | 1 | 1 | 1 | 1 | 1 | 1 | 1 | 1 | 88% |
| Q4 | 1 | 1 | 0 | 1 | 1 | 1 | 1 | 1 | 0 | 0 | 0 | 1 | 1 | 1 | 1 | 1 | 0 | 71% |
| Q5 | 1 | 1 | 1 | 1 | 1 | 1 | 1 | 1 | 1 | 1 | 1 | 1 | 1 | 1 | 1 | 1 | 1 | 100% |
| Q6 | 1 | 1 | 1 | 1 | 1 | 1 | 1 | 1 | 1 | 1 | 1 | 1 | 1 | 1 | 1 | 1 | 1 | 100% |
| Q7 | 1 | 1 | 1 | 1 | 1 | 1 | 1 | 0 | 1 | 1 | 0 | 1 | 1 | 1 | 1 | 1 | 1 | 88% |
| Q8 | 1 | 0 | 1 | 0 | 1 | 0 | 0 | 1 | 1 | 0 | 1 | 0 | 1 | 1 | 0 | 0 | 0 | 47% |
| Q9 | 1 | 1 | 1 | 0 | 1 | 1 | 1 | 1 | 1 | 1 | 1 | 1 | 1 | 1 | 1 | 1 | 1 | 94% |
| Q10 | 1 | 1 | 0 | 0 | 0 | 1 | 0 | 0 | 0 | 0 | 0 | 1 | 1 | 0 | 0 | 1 | 1 | 41% |
| Q11 | 0 | 0 | 0 | 0 | 0 | 0 | 0 | 0 | 0 | 0 | 1 | 0 | 0 | 0 | 0 | 0 | 0 | 6% |
| Q12 | 1 | 1 | 0 | 0 | 1 | 0 | 1 | 0 | 0 | 0 | 0 | 1 | 0 | 1 | 0 | 0 | 1 | 41% |
| Q13 | 1 | 1 | 1 | 0 | 0 | 1 | 0 | 0 | 1 | 1 | 0 | 1 | 0 | 0 | 0 | 1 | 0 | 47% |
| Q14 | 1 | 1 | 1 | 1 | 1 | 0 | 0 | 0 | 1 | 1 | 0 | 1 | 0 | 1 | 1 | 1 | 1 | 71% |
| Q15 | 0 | 0 | 0 | 0 | 0 | 0 | 0 | 0 | 0 | 0 | 0 | 0 | 0 | 0 | 0 | 0 | 0 | 0% |
| Q16 | 0 | 0 | 1 | 0 | 0 | 0 | 0 | 0 | 1 | 1 | 0 | 1 | 0 | 0 | 1 | 1 | 1 | 41% |
| Q17 | 0 | 0 | 0 | 0 | 0 | 0 | 0 | 0 | 1 | 0 | 0 | 0 | 0 | 0 | 0 | 0 | 0 | 6% |
| Q18 | 0 | 1 | 1 | 1 | 0 | 0 | 0 | 1 | 1 | 1 | 0 | 1 | 1 | 1 | 1 | 1 | 1 | 71% |
| Q19 | 0 | 0 | 1 | 1 | 1 | 1 | 1 | 1 | 1 | 1 | 0 | 1 | 1 | 1 | 1 | 1 | 1 | 82% |
| Q20 | 0 | 0 | 0 | 0 | 1 | 0 | 1 | 0 | 1 | 0 | 0 | 1 | 1 | 1 | 1 | 1 | 1 | 53% |
| Q21 | 0 | 1 | 1 | 1 | 1 | 1 | 1 | 1 | 1 | 1 | 0 | 1 | 1 | 1 | 1 | 1 | 0 | 82% |
| Q22 | 1 | 0 | 1 | 0 | 0 | 1 | 0 | 0 | 0 | 1 | 1 | 0 | 0 | 0 | 0 | 0 | 0 | 29% |
| Q23 | 0 | 0 | 0 | 0 | 0 | 0 | 0 | 0 | 0 | 0 | 0 | 0 | 0 | 0 | 0 | 0 | 0 | 0% |
| Q24 | 1 | 0 | 1 | 0 | 0 | 1 | 0 | 0 | 0 | 1 | 0 | 0 | 0 | 0 | 0 | 0 | 0 | 24% |
| Q25 | 0 | 0 | 0 | 0 | 0 | 0 | 0 | 0 | 0 | 0 | 0 | 0 | 0 | 0 | 0 | 0 | 0 | 0% |
| Q26 | 1 | 1 | 1 | 1 | 0 | 1 | 1 | 0 | 1 | 1 | 1 | 1 | 1 | 1 | 1 | 1 | 1 | 88% |
| Q27 | 0 | 0 | 1 | 0 | 0 | 1 | 0 | 1 | 1 | 1 | 0 | 0 | 1 | 1 | 0 | 0 | 0 | 41% |
| Q28 | 0 | 0 | 1 | 0 | 0 | 1 | 0 | 1 | 1 | 1 | 0 | 0 | 0 | 0 | 0 | 0 | 0 | 29% |
| Q29 | 0 | 1 | 0 | 1 | 0 | 0 | 1 | 1 | 0 | 0 | 0 | 1 | 0 | 0 | 0 | 0 | 0 | 29% |
| Q30 | 0 | 1 | 0 | 0 | 0 | 0 | 1 | 1 | 0 | 0 | 0 | 1 | 0 | 0 | 0 | 0 | 0 | 24% |
| Q31 | 0 | 1 | 0 | 0 | 1 | 0 | 0 | 0 | 0 | 0 | 0 | 1 | 0 | 0 | 1 | 1 | 1 | 35% |
| Q32 | 0 | 1 | 0 | 0 | 1 | 0 | 1 | 1 | 0 | 0 | 0 | 1 | 0 | 0 | 0 | 0 | 0 | 29% |
| Q33 | 1 | 1 | 0 | 1 | 0 | 0 | 1 | 1 | 0 | 1 | 0 | 1 | 0 | 1 | 1 | 1 | 1 | 65% |
| Q34 | 0 | 0 | 0 | 0 | 0 | 0 | 0 | 1 | 0 | 1 | 0 | 0 | 1 | 1 | 1 | 1 | 0 | 35% |
| Q35 | 0 | 0 | 0 | 1 | 1 | 0 | 1 | 1 | 0 | 1 | 0 | 0 | 0 | 1 | 1 | 1 | 1 | 53% |
| Q36 | 0 | 0 | 0 | 0 | 0 | 0 | 1 | 1 | 0 | 0 | 0 | 1 | 1 | 0 | 1 | 0 | 0 | 29% |
| Q37 | 1 | 1 | 1 | 1 | 1 | 1 | 1 | 1 | 1 | 1 | 1 | 1 | 1 | 1 | 1 | 1 | 1 | 100% |
| % | 49% | 57% | 49% | 43% | 49% | 49% | 54% | 54% | 49% | 57% | 24% | 65% | 49% | 57% | 54% | 57% | 49% |  |

**Table S2.** Fracture type, implant/surgery specifications, and load case of the finite element simulation of the reviewed articles [1-17].

| **Article** | **Fracture Type** | **Implant/Surgery Spec.^ξ^** | **Load case** |
| --- | --- | --- | --- |
| Calişal and Uğur [1] | Midshaft, transverse, 1-mm gap | 2.5-mm 6-hole anatomical LCP (L: 82, D_h_: 3.5, D_s_: 2.4); anterior and superior placement. | Shoulder abduction & flexion, 0° to 150° in 1 sec under G = 9806.6mms^-1^. |
| Cheng, et al. [2] | Midshaft, transverse, 0.5-mm gap | 3D printed 7-hole 3.5-mm anterior LCP (L: 104, W: 10, D_s_: 3.5). | 100 N inferior bending & 100 N inferior bending with axial comp. at distal end. |
| Cronskär, et al. [3] | Midshaft, transverse, 0.5-mm gap | 6-hole 3.5-mm pre-contoured recon. LCP. | Apply sternum & acromion contact force & distributed forces at the attachment of SCM, DT, TPZ, CL, estimated by MSK model. |
| Han, et al. [4] | Midshaft, transverse, 4-mm gap | 70-hole LCP (D_s_: 3.5) with and without groove and berried central hole. | 100 N axial comp., inferior bending, 1 Nm axial torque at distal end, the combination of these three. |
| Huang, et al. [6] | Midshaft, transverse, 2.5-mm gap | 8-hole 2.0-mm conceptual anterior LCP, superior LCP, spiral LCP (D_h_: 4.0, D_s_: 2.4) | 100 N axial comp., inferior bending, 1 Nm axial torque at distal end. |
| Huang, et al. [5] | Distal, transverse (20 mm from AC joint) | Hook LCP, superior single LCP, dual LCP. | Apply distributed forces at the attachment of SCM & TPZ. Both ends fixed. |
| Kim, et al. [7] | Midshaft, comminute, 10-mm gap | 2.5-mm 7-hole superior LCP with and without screw cap | 100 N axial comp., inferior bending, 1 Nm axial torque at distal end. |
| Kim, et al. [8] | Midshaft, transverse | Anatomical LCP (baseline), with berried central hole, with double-curved wing shape. | 100 N axial comp., inferior bending, 1 Nm axial torque at distal end. |
| Kritsaneephaiboon, et al. [9] | Proximal (25 mm from SC joint), transverse and comminute, 2-mm and 10-mm | Inverted lateral clavicle LCP, anatomical superior LCP, reconstruction LCP (D_s_: 2.7 and 3.5) | 100 N axial comp., inferior bending, 1 Nm axial torque at distal end. |
| Marie [10] | Midshaft, oblique (38°) | Recon. LCP (anterior (or anteoinferior) and superior, with and without lag screws, and with lag screw through plate), pre-contoured superoanterior LCP (with and without lag screw) | Apply sternum & acromion contact force and distributed forces at the attachment of SCM, DT, TPZ, CL, estimated by MSK model. |
| Marinescu, et al. [11] | Midshaft, transverse | Anatomical recon. LCP. | DT & PEA muscle force with 10N, 50N, 100N, 140N. |
| Ni, et al. [12] | Midshaft, transverse, 0.5-mm gap | 3.5-mm LCP (D_s_: 3.5), Sonoma intramedullary nail, Rockwood clavicle pin. | 100 N axial comp., inferior bending, 1 Nm axial torque at distal end. |
| Ni, et al. [13] | Midshaft, type 15-B2 comminute with butterfly segment | 3.5-mm 8-hole superior LCP (with double inner cerclage wirings, double outer cerclage wiring, single interfragmentary screw, and double interfragmentary screws) (D_s_: 3.5, D_w_: 1.0). | 100 N axial comp., inferior bending, 1 Nm axial torque at distal end. |
| Wu, et al. [14] | Midshaft, oblique (15-B1.2), 2.5-mm gap | 3.0-mm superior anterior LCP with lateral expansion (L: 108, D_s_: 2.7 and 3.5, D_ns_: 4.0), 3.0-mm Aplus C.A.S. LCP system (L: 110, D_s_: 2.7 and 3.5) | 200 N axial comp., inferior bending, 4 Nm axial torque at distal end. |
| Zeng, et al. [15] | Midshaft, transverse, 1-mm gap | 3.5-mm 7-hole recon. plate (D_s_: 3.5), titanium elastic nail (D: 2.5) | 250 N axial comp., inferior bending at distal end. |
| Zhang, et al. [16] 2020 | Midshaft, transverse, 4-mm gap | 3.3-mm 6-hole pre-contoured large bend superior recon. plate (L: 98, W: 11, D_s_: 3.5), anteroinferior recon. plate (L: 95, W: 11, D_s_: 3.5), 1.6-mm 6-hole dual small plating (L:85, W:11) | 100 N axial comp., inferior bending, 1 Nm axial torque at distal end. |
| Zhang, et al. [17] | Midshaft, transverse, 4-mm gap | 7-hole spiral LCP with locking screws (D: 3.5, D_s_: 3.5), with Herbert screw (L: 85, D: 4.5) | 100 N axial comp., inferior bending, 1 Nm axial torque at distal end. |

AC: acromioclavicular; comp.: compression; CL: conoid ligament; DT: deltoid; MSK: musculoskeletal; PEA: pectoralis anterior; recon.: reconstruction; SCM: sternocleidomastoid; SC: sternoclavicular; TPZ: trapezius; spec.: specifications.

^ξ^D: diameter; D_h_: hole diameter; D_ns_: non-locking cortex screw diameter; D_s_: screw diameter; D_w_: wiring diameter; L: length; LCP: locking plate; W: width (all dimension in mm)

**Table S3.** Independent (factors of interest) and dependent factors (outcome measures) of the reviewed articles [1-17].

| **Article** | **Independent factor(s)** | **Level(s) of factor(s) of interest** | **Variable(s) of interest** |
| --- | --- | --- | --- |
| Calişal and Uğur [1] | Implant | 1. Ant. placement 2. Sup. placement | - VMS: ligament, joints, ligament, plate, screws, bone - Life span |
|  | Load Case | 1. Shoulder abduction 2. Shoulder flexion |  |
| Cheng, et al. [2] | Implant | 1. Intact 2. Stainless steel implant 3. Titanium alloy implant 4. Magnesium alloy implant | - Structural stiffness - Plate stiffness - VMS of plate - Strain at fracture site |
| Cronskär, et al. [3] | Implant | 1. Intact 2. Fixation | - VMS: bone and plate - Displacement: plate |
| Han, et al. [4] | Implant | 1. Original spiral LCP 2. Modified spiral LCP by removing the groove and filling the hole near the fracture | VMS: bone and plate |
|  | Load case | 1. Axial compression 2. Inf. bending 3. Axial torsion 4. Combination of 1 to 3 |  |
| Huang, et al. [6] | Implant | 1. Ant. clavicle plate 2. Sup. clavicle plate 3. Spiral clavicle plate | - Structural stiffness - VMS: screws and plate |
|  | Load case | 1. Axial compression 2. Inf. bending 3. Axial torsion |  |
| Huang, et al. [5] | Implant | 1. Hook plate 2. Sup. single plate 3. Dual plate | - VMS: bone, plate, and screws - Displacement: bone |
| Kim, et al. [7] | Screws cap | 1. LCP w/ screw cap 2. LCP w/o screw cap | VMS: plate at each hole |
|  | Load case | 1. Axial compression 2. Inf. bending 3. Axial torsion |  |
| Kim, et al. [8] | Implant | 1. LCP 2. LCP w/ berried central hole 3. LCP w/ double-curved wing | VMS: plate (at each hole) |
|  | Load case | 1. Axial compression 2. Inf. bending 3. Axial torsion |  |
| Kritsaneephaiboon, et al. [9] | Implant | 1. Inverted (lateral clavicle) LCP 2. Anatomical superior LCP 3. Dual plating (anatomical sup. + recon.) LCP | - VMS: bone and implant - Elastic strain: bone fracture site |
|  | Load case | 1. Axial compression 2. Inf. bending 3. Axial torsion |  |
| Marie [10] | Implant | 1. Ant. LCP recon. w/o lag screw 2. Ant. LCP recon. w/ lag screw 3. Sup. LCP recon. w/o lag screw 4. Sup. LCP recon. w/ lag screw 5. Sup. LCP recon. w/ lag screw thru plate 6. Sup.ant. LCP recon. w/o lag screw 7. Sup.ant. LCP recon. w/ lag screw | - VMS: bone and plate - Displacement: plate - Max principal strain: plate |
| Marinescu, et al. [11] | Load case | 1. Deltoids activation 2. Pectoralis ant. activation | - VMS: bone and implant - Deformation: bone and implant |
|  | Load | 10 N, 50 N, 100 N, 140 N |  |
| Ni, et al. [12] | Implant | 1. LCP 2. Anatomical intramedullary nail 3. Straight intramedullary nail | - Construct stiffness - Micromotion: fracture site - VMS: bone and implant |
|  | Load case | 1. Axial compression 2. Inf. bending |  |
| Ni, et al. [13] | Implant | 1. LCP w/ double inner cerclage wirings 2. LCP w/ double outer cerclage wirings 3. LCP w/ single interfragmentary screws 4. LCP w/ double interfragmentary screws | - VMS: bone and implant - Micromotion: fracture site |
|  | Load case | 1. Axial compression 2. Inf. bending |  |
| Wu, et al. [14] | Implant | 1. Intact 2. Sup.ant. Spiral LCP 3. C.A.S. LCP | - Structural stiffness - VMS: plate |
| Zeng, et al. [15] | Implant | 1. Titanium elastic nail 2. Recon. plate | Displacement of distal clavicle, VMS of bone and implant |
|  | Load case | 1. Axial compression 2. Inf. bending |  |
| Zhang, et al. [16] | Implant | 1. Sup. plate 2. Ant.inf. plate 3. Dual plate | VMS, and displacement of models and implant, structural stiffness, micro-motions |
|  | Load case | 1. Axial compression 2. Inferior bending 3. Axial torsion |  |
| Zhang, et al. [17] | Implant | 1. Spiral LCP 2. Herbert screw | Structural stiffness, VMS of bone and implant, micro-motion |
|  | Load case | 1. Axial compression 2. Inferior bending 3. Axial torsion |  |

ant.: anterior; inf.: inferior; LCP: locking plate; recon.: reconstruction; sup.: superior; VMS: von Mises stress; w/: with; w/o: without.

**References**

[1] E. Calişal, L. Uğur. 2018. Evaluation of the plate location used in clavicle fractures during shoulder abduction and flexion movements: A finite element analysis. Acta of bioengineering and biomechanics. 20(4): 41-6.

[2] R. Cheng, Z. Jiang, D. Dimitriou, W. Gong, T.Y. Tsai. 2021. Biomechanical analysis of personalised 3d-printed clavicle plates of different materials to treat midshaft clavicle fractures. Journal of Shanghai Jiaotong University (Science). 26(3): 259-66.

[3] M. Cronskär, J. Rasmussen, M. Tinnsten. 2015. Combined finite element and multibody musculoskeletal investigation of a fractured clavicle with reconstruction plate. Computer Methods in Biomechanics and Biomedical Engineering. 18(7): 740-8.

[4] Z.H. Han, Q. Luo, G.Y. Deng, C. Bi, G. Yin, H.D. Lin et al. 2023. Canceling notch improves the mechanical safety of clavicle locking plate: A 3d finite element study. Orthopaedic Surgery. 15(8): 2152-6.

[5] D.Q. Huang, Z.Q. Hu, W.L. Feng, X.P. Wang. 2023. Dual plate has better biomechanical stability than hook plate or superior single plate for the fixation of unstable distal clavicle fractures: A finite element analysis. Archives of Orthopaedic and Trauma Surgery. 143(8): 4805-12.

[6] T.L. Huang, W.C. Chen, K.J. Lin, C.L. Tsai, K.P. Lin, H.W. Wei. 2016. Conceptual finite element study for comparison among superior, anterior, and spiral clavicle plate fixations for midshaft clavicle fracture. Medical Engineering & Physics. 38(10): 1070-5.

[7] D.-G. Kim, S. Min Kim, Y. Kim. 2022. Numerical simulation and biomechanical analysis of locking screw caps on clavicle locking plates. Medicine. 101(30): e29319-e.

[8] S.M. Kim, S.T. Kim, D.W. Han, D.G. Kim. 2024. Study on the characteristics of increased mechanical stiffness according to changes in lcp shape to reinforce clavicle fractures. Scientific reports. 14(1): 6382.

[9] A. Kritsaneephaiboon, S. Jitprapaikulsarn, N. Chantarapanich, P. Klabklay, A. Gromprasit, C. Patamamongkonchai et al. 2024. The application of inverted lateral clavicle locking compression plate for medial clavicle fractures: Biomechanical study and clinical implementation. European Journal of Orthopaedic Surgery and Traumatology. 34(3): 1465-78.

[10] C. Marie. 2015. Strength analysis of clavicle fracture fixation devices and fixation techniques using finite element analysis with musculoskeletal force input. Medical & Biological Engineering & Computing. 53(8): 759-69.

[11] R. Marinescu, V.I. Antoniac, D.I. Stoia, D.C. Lăptoiu. 2017. Clavicle anatomical osteosynthesis plate breakage – failure analysis report based on patient morphological parameters. Romanian Journal of Morphology and Embryology. 58(2): 593-8.

[12] M. Ni, W. Niu, D.W.-C. Wong, W. Zeng, J. Mei, M. Zhang. 2016. Finite element analysis of locking plate and two types of intramedullary nails for treating mid-shaft clavicle fractures. Injury. 47(8): 1618-23.

[13] M. Ni, F. Zhang, J. Mei, C.Y.J. Lin, S.M.S. Gruber, W. Niu et al. 2020. Biomechanical analysis of four augmented fixations of plate osteosynthesis for comminuted mid‑shaft clavicle fracture: A finite element approach. Experimental and Therapeutic Medicine. 20(3): 2106-12.

[14] D.K. Wu, W.C. Chen, K.J. Lin, C.L. Tsai, K.P. Lin, H.W. Wei. 2021. Advantage of multi-directional stability of spiral clavicle plate in treatment of middle one-third clavicle fracture: A finite element study. Journal of Medical and Biological Engineering. 41(4): 405-11.

[15] L. Zeng, H. Wei, Y. Liu, W. Zhang, Y. Pan, W. Zhang et al. 2015. Titanium elastic nail (ten) versus reconstruction plate repair of midshaft clavicular fractures: A finite element study. PLoS One. 10(5): e0126131.

[16] F. Zhang, F. Chen, Y. Qi, Z. Qian, S. Ni, Z. Zhong et al. 2020. Finite element analysis of dual small plate fixation and single plate fixation for treatment of midshaft clavicle fractures. Journal of Orthopaedic Surgery and Research. 15(1): 148.

[17] X.J. Zhang, X.D. Cheng, B. Yin, J.Z. Wang, S. Li, G.B. Liu et al. 2019. Finite element analysis of spiral plate and herbert screw fixation for treatment of midshaft clavicle fractures. Medicine. 98(34).
